# Scalar model for frictional precursors dynamics

**DOI:** 10.1038/srep08086

**Published:** 2015-02-02

**Authors:** Alessandro Taloni, Andrea Benassi, Stefan Sandfeld, Stefano Zapperi

**Affiliations:** 1 CNR-IENI, Via R. Cozzi 53, 20125 Milano, Italy; 2 Empa, Swiss Federal Laboratories for Materials Science and Technology, CH-8600 Dübendorf, Switzerland; 3 Institute of Materials Simulation (WW8), Department of Materials Science, University of Erlangen-Nürnberg (FAU), Dr.-Mack-Str. 77, 90762 Fürth, Germany; 4 ISI Foundation, Via Alassio 11/C 10126 Torino, Italy; 5 Department of Applied Physics, Aalto University, P.O. Box 14100, FIN-00076, Aalto, Finland

## Abstract

Recent experiments indicate that frictional sliding occurs by nucleation of detachment fronts at the contact interface that may appear well before the onset of global sliding. This intriguing precursory activity is not accounted for by traditional friction theories but is extremely important for friction dominated geophysical phenomena as earthquakes, landslides or avalanches. Here we simulate the onset of slip of a three dimensional elastic body resting on a surface and show that experimentally observed frictional precursors depend in a complex non-universal way on the sample geometry and loading conditions. Our model satisfies Archard's law and Amontons' first and second laws, reproducing with remarkable precision the real contact area dynamics, the precursors' envelope dynamics prior to sliding, and the normal and shear internal stress distributions close to the interfacial surface. Moreover, it allows to assess which features can be attributed to the elastic equilibrium, and which are attributed to the out-of-equilibrium dynamics, suggesting that precursory activity is an intrinsically quasi-static physical process. A direct calculation of the evolution of the Coulomb stress before and during precursors nucleation shows large variations across the sample, explaining why earthquake forecasting methods based only on accumulated slip and Coulomb stress monitoring are often ineffective.

The classical laws of friction, due to Amontons and Coulomb, postulate that a body resting on a surface can be displaced only by applying a shear force larger than a static friction force, which is proportional to the normal load and independent of the apparent area of contact. Recent research has challenged this understanding of friction, showing that macroscopic slip is due to the formation and propagation of detachment fronts through the contact interface^1^. The nature of these fronts and their speed depend on the way shear is applied to the sample and on its geometry^2^,^3^, a particularly compelling issue in view of the long held assumption of independence of friction on the sample shape and size. It is particularly intriguing that, in some cases, localized sliding precursors nucleate long before the applied force reaches the static friction force at which the front propagates through the entire contact interface^1^,^4^,^5^. Numerical simulations of friction models in one^6^,^7^,^8^,^9^,^10^ and two dimensions^11^,^12^,^13^ allow to study the main features of the spatio-temporal dynamics of precursors. These numerical works have mainly focused on the qualitative dynamical aspects of propagation, reproducing the different dynamical regimes observed in experiments^6^,^7^,^8^,^9^ and the nucleation of the fronts under various loading conditions^11^.

Based on the results of experiments^4^ and numerical simulations^11^ it was suggested that frictional precursors evolve according to universal laws: the sample size and normal load dependences of precursors lengths can be rescaled away and different experiments can be collapsed a single master curve. Establishing universal forms for slip precursors would be particularly important for earthquake forecasting^14^. Slip or stress accumulation on faults has been often observed to accelerate close to large earthquakes^15^,^16^,^17^, but detailed predictions based on this are considered to be unreliable^18^,^19^. It is therefore extremely important to better clarify the conditions leading to precursors and confirm their universality. Another puzzling aspect revealed by experiments is an apparent violation of the Amontons-Coulomb laws: direct measurement of shear *τ* and normal stresses *σ* close to the frictional interface indicated regions where the Coulomb stress *τ_C_* = |*τ*| − *μ*|*σ*| is positive without inducing detachment^2^,^3^. This result suggests that the friction coefficient *μ* might not be a well defined material constant as conventionally assumed.

Scalar models are commonly used to study the planar crack front propagation in disodered elastic media^20^,^21^, in quasi-two dimensional geometries^22^ and under antiplane shearing conditions^23^. On the other hand, recent experiments have provided the evidence that classical shear cracks singular solutions, originally devised to account for brittle fractures, offer a quantitative excellent description of the static-to-dynamic friction transition^24^. Here, combining the solution of three dimensional scalar elastic equations in finite geometries with simple contact mechanical rules for local slip at the frictional interface, we reproduce accurately the experimentally observed evolution of the contact area as the sample is loaded. In this way, we obtain a complete picture of the role played by sample geometry and loading conditions on the precursors nucleation. Moreover we show that precursors originate from stress gradients on the contact interface and are therefore absent when loading is applied uniformly through the top of the slider. Disorder induced precursors nucleation of the kind predicted for sliding thin films and monolayers^25^ should be strongly suppressed in three dimensions due to long-range elastic interactions which make the coherence length extremely large^26^. When shear is applied on the slider side, however, we observe precursors whose evolution depends in a non-universal way on the sample geometry. The occurrence of universal profiles is explained by the symmetries of the interfacial shear stress obtained analytically.

Despite its quasi-static nature, our model incorporates the Achard's law and the Amontons' first and second laws, reproducing several key features observed in experiments including the discrete stress drops observed in correspondence of the slip precursors. Most importantly, our model can help to assess which experimental feature can be attributed to the static elastic equilibrium, and which instead is a pure dynamical out-of-equilibrium aspect. Our calculations reproduce the experimental interfacial stress profiles detected at the frictional interface, before slip. We discuss the large fluctuations of the internal stresses during precursors activity in the bulk of the material, and we provide the numerical and analytical evidence of this large heterogeinity. This observation, substantiated also by finite element model simulations, suggests that drawing firm conclusions based on the value of the Coulomb stress measured away from the contact interface, both in laboratory experiments and in earthquake faults, could be problematic.

## Results

### Simulations for different sample sizes and loading conditions

**Figure 1:**
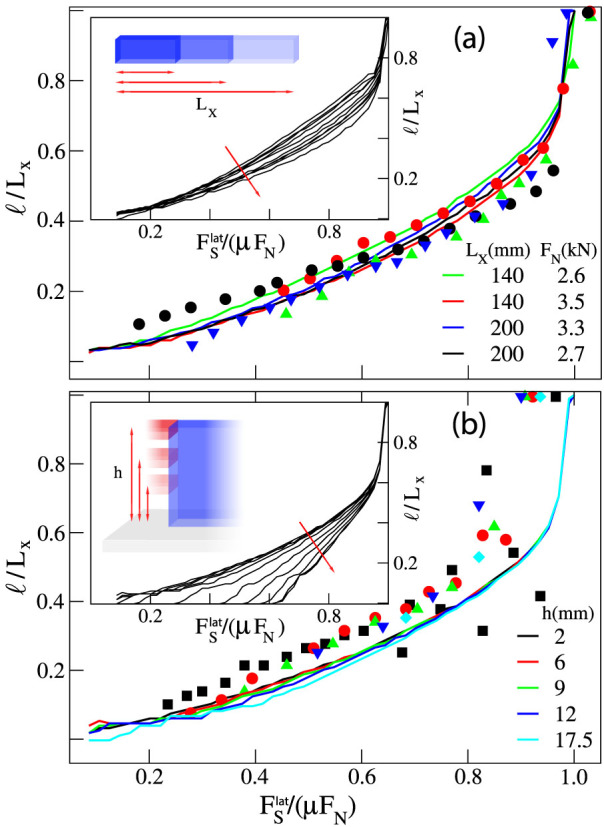
The dependence of slip precursors on the sample size and the point of application of the lateral force. (a) Experimental data (symbols) from Ref. 4 obtained for different *L_x_* and *F_N_* show an approximate data collapse when the rescaled front position * */*L_x_* is plotted against the rescaled lateral shearing force 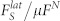 (*L_y_* = 7 mm, *L_z_* = 75 mm, *h* = 6 mm). This result is accurately reproduced by our model (solid lines). Inset shows that when the range of *L_x_* is increased further, collapse is lost (100 *mm* < *L_x_* < 350 mm). (b) Similarly, experimental data (symbols) from Ref. 4 indicate that the rescaled precursors profiles are approximately independent of the height *h* (the point of application of the lateral force 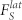), in perfect agreement with the numerical outcomes (solid lines). *L_x_* = 140 mm, *L_y_* = 7 mm, *L_z_* = 75 mm, *F_N_* = 3 kN. In the inset, we show that no collapse arises if *h* is increased beyond the experimental values (2 *mm* < *h* < 73 mm).

Following Ref. 4, we first study how precursors depend on *L_x_* and on the normal load *F_N_* when 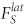 is applied through a rod placed on the trailing edge, at height *h* = 6 mm. Experimental evidence suggests that the precursor size *ℓ* obtained for different values of *L_x_* and *F_N_* can be collapsed into a single master curve when normalized by *L_x_* and plotted versus 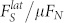. Our numerical results reproduce quantitatively the experimental findings as shown in Fig. 1(a). In our model, however, we are able to change *L_x_* over a wider range than in the experiments, revealing that data collapse is in fact only approximate (see inset of Fig. 1(a)). Similar behavior is obtained when we vary *L_y_* (Fig. S4) or *L_z_* (Fig. S5) keeping constant the other parameters: in all cases front precursors exhibit a dependence on the sample dimensions. We have also changed *L_x_* and *L_y_* holding their ratio *L_x_*/*L_y_* unchanged (Fig. S6), *L_x_* and *L_z_* with *L_x_*/*L_z_* constant (Fig. S7), or *L_y_* and *L_z_* with *L_y_*/*L_z_* constant (Fig. S8). Again, data collapse is not obtained, indicating that for this loading condition the precursor lengt *ℓ* depends in a non-trivial way on the sample dimensions (*L_x_*, *L_y_*, *L_z_*). The general trend however is that precursory activity tends to decrease as the varying dimension is increased: for a larger sample we typically need a larger shear force to observe a precursor of a given length.

Experimental results in Ref. 4 also suggest that the height *h* at which the lateral force is applied to the sample trailing edge has no influence on the evolution of the front precursors. While this is true for the range of *h* used in experiments (see Fig. 1(b)), when we increase *h* further the curves no longer collapse. In particular, we find that the lateral force needed to nucleate the first precursor increases considerably with *h* (see the inset of Fig. 1(b)). Remarkably, this effect persists when we increase both *h* and *L_z_* leaving their ratio constant (Fig. S9). Yet, experiments have provided the evidence that the precursors length *ℓ* advances by periodical discrete leaps of roughly equal size, which take place at nearly constant increments of *F_S_*. Moreover, this periodicity exhibits an apparent scaling with *h*, becoming larger with increasing *h*^27^. While the envelope of the curves reproduced by our model do show periodicity in the increments of the precursors sizes, the size of these discrete jumps and the corresponding increments of *F_S_* seem to remain unaffected by varying *h*, at least within the range of heights used in the experiments.

**Figure 2:**
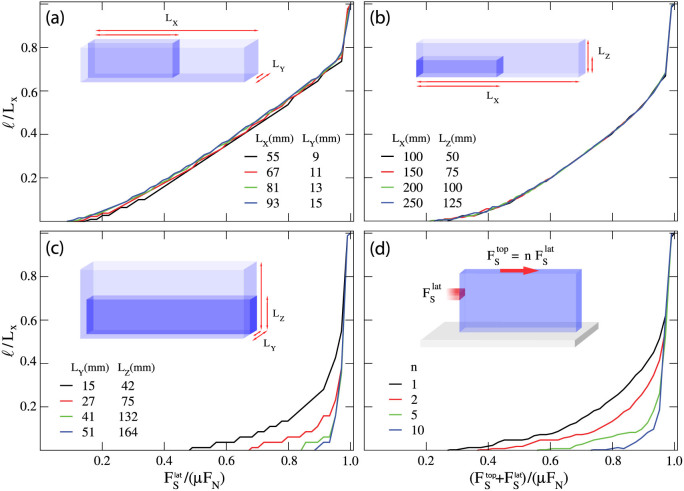
The dependence of slip precursors on the sample aspect ratios and on the loading conditions. Rescaled precursors quasi-static evolution obtained when an uniform side shear 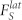 is applied. (a) Curves exhibit the same universal behavior for different *L_x_* and *L_y_* but same aspect ratio 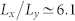, with *F_N_* = 4 kN and *L_z_* = 10 mm. (b) Perfect collapse of the curves is obtained when the aspect ratio *L_x_*/*L_z_* and *L_y_* are kept constant. 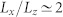, *L_y_* = 7 mm, *F_N_* = 4 kN. (c) Precursors progressively disappear when *L_y_* and *L_z_* are increased by leaving unchanged the ratio *L_y_*/*L_z_*, and *L_x_* is constant. 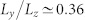, *L_x_* = 40 mm, *F_N_* = 4 kN. This findings are consistent with the assumption that precursors evolution profiles reflect the same simmetries appearing in the shear stress at the frictional interface S97, which is function of the quantity 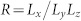. (d) While simultaneously loading the sample from top and from the edge with a rod (*h* = 6 mm and  *_h_* = 2 mm), 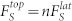, precursors dynamics is suppressed for large *n*. Sample parameters are *F_N_* = 2.7 kN, *L_x_* = 201 mm, *L_y_* = 7 mm *L_z_* = 132 mm.

We explore further the dependence of precursors on the sample geometry by considering a different loading condition in which the lateral force is applied uniformly on the sample side surface (2Δ*h* = *L_z_*). In this case, we find that the precursors are size independent when we vary *L_x_* and *L_y_* keeping their ratio *L_x_*/*L_y_* constant (Fig. 2(a)) or *L_x_* and *L_z_* with constant *L_x_*/*L_z_* (Fig. 2(b)). When we vary instead *L_y_* and *L_z_*, keeping constant *L_y_*/*L_z_*, no universality is found and precursors again tend to disappear for large sample sizes (Fig. 2(c)). A similar effect is obtained using mixed mode loading as in Refs. 2, 3, applying simultaneously a shear force on the top surface and on the trailing edge. As the ratio between both forces 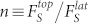 increases, the length of the precursor shrinks (Fig. 2(d)) and disappear when loading is only applied on the sample top plate.

Precursors are defined by detecting the decay of the real area of contact. This feature is perfectly reproduced by our model, and, roughly speaking, it is ultimately due to the detachment of regions of the frictional interface satisfying the local static friction condition (19). Thus, the dependence of the precursor envelope profile on the sample geometry should reflect the properties of the Coulomb stress across the entire contact interface. In the Supplementary Information (sec. IX), we discuss how some general aspects of the precursor shape can be deduced from the symmetry of the Green function.

### Normal and shear stresses at the interface and the Amontons law

Direct experimental measurements of shear and normal stress profiles close to the contact interface show that the Coulomb stress can exceed zero locally, without inducing any detachment front, precursor or local slip^2^,^3^. This is puzzling since it would represent a local violation of the Amontons-Coulomb law, suggesting that the friction coefficient might not be a material constant. In our model, however, the local and global friction coefficient *μ* is fixed across the whole interface, and local detachment occurs if *τ_C_*(*x*, *y*, 0) > 0 by construction (Eqs.(19) and (S96)). Yet, this apparent contradiction can be resolved by noting that local stresses in Refs. 2, 3 are measured on a reference plane located at a height of *z_P_* = 2 mm above the frictional interface.

**Figure 3:**
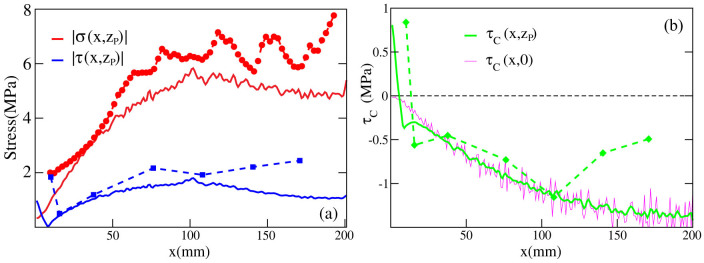
Coulomb stress as slip precursor. (a) Normal (red) and shear stress (blu) averaged over the *y* direction. Profiles of 
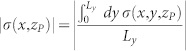
 and 
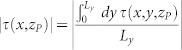
 across the sample length are calculated on a reference plane *z_P_* = 2 mm above the frictional interface (solid lines) and compared with the experimental data from Ref. 2 (Fig. 2A, slow front) (symbols-dashed lines). Shear force was applied on top as well as on the sample trailing edge according to the experimental setup (Ref. 2). Stress calculation was performed at a value of the shear force *F_S_* right before the nucleation of the precursor (see Fig. S10 and Fig. 8(a)). *L_x_* = 200 mm, *L_y_* = 7 mm, *L_z_* = 100 mm, *F_N_* = 6.25 kN. (b) Coulomb stress calculated on the reference plane above the contact interface and averaged over the *y* direction (solid green line): * _C_*(*x*, *z_P_*) = |* *(*x*, *z_P_*)|   * *|* *(*x*, *z_P_*)|. Comparison with data inferred from Ref. 2 is excellent (green symbols-dashed line). The magenta line represents the *y*-averaged Coulomb stress at the frictional interface 
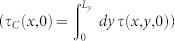
: although no detachment front is yet present at the contact plane (* _C_*(*x*, 0) < 0 throughout the surface), the value of the Coulomb stress at the reference plane *z_P_* = 2 mm can exceed locally the threshold, leading to the erroneous conclusion that Amonton-Coulomb law might be violated.

Thanks to the analytical solvability of our model we can compute the shear and normal stresses at any points (*x*, *y*, *z*) of the slider bulk: this is perfomed in the Suppl. Mat. sec. VIII (see Eqs.(S71), (S81) or Eqs.(S86),(S89)). Calculating the stresses on the plane *z_P_* = 2 mm yields a good quantitative agreement with experiments (Fig. 3(a), S10). In particular, the curves shown in Fig. 3 represent the shear and normal stresses averaged over the *y* direction (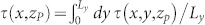 and 
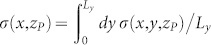
), along the entire sample 0 < *x* < *L_x_*, just before the onset of the first precursor, i.e. when no detachment is yet present at the contact interface. This can be seen also from Fig. S10 where the full quasi-static dynamics of *τ*(*x*, *z_P_*) and *σ*(*x*, *z_P_*) are plotted. The corresponding Coulomb stress on the same plane (*τ_C_*(*x*, *z_P_*) = |*τ*(*x*, *z_P_*)| − *μ*|*σ*(*x*, *z_P_*)| or 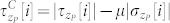 from Eq.(S91)) prior to the nucleation of the first precursor event is reported in Fig. 3(b) (solid green line) showing again a good agreement with the experimental data.

**Figure 4:**
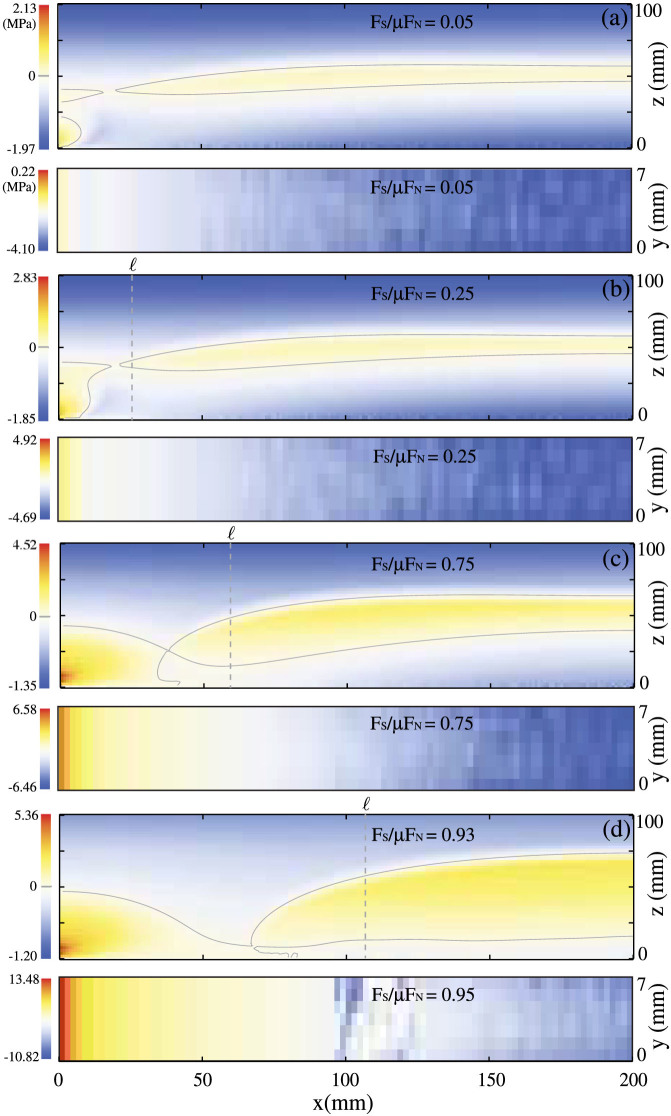
Full Coulomb stress at the frictional interface. (a)–(d) Quasi-static evolution of the Coulomb stress (averaged over *y*) along the slab *x* – *z* plane (on top), and on the plane *z* = *z_P_* (bottom panels). Color code indicates regions where * _C_* > 0 (yellow–red) from those for which * _C_* < 0 (blu), grey solid lines correspond to the set of points fulfilling * _C_* = 0. Panel (a) refers to the slider sitution before the first precursor event nucleates, the plane *z* = *z_P_* = 2 mm (bottom panels) is where quantities in Fig. 7 are calculated (see also Fig. S10 dashed black lines). Grey dashed lines represent the precursor envelope * * at the frictional interface, obtained from the real contact area decay (see Fig. 2(b)(inset) and Fig. 3).

Our result may suggest the observed apparent violation of the Amontons first law^2^,^3^ could be due to the fluctuation undergone by the internal stresses in the material bulk, even in the vicinity (but not on) the slider frictional interface. Defining a local friction coefficient as the ratio 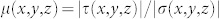, is not an eligible procedure if the point (*x*, *y*, *z*) does not lie on the frictional plane (*x*, *y*, 0). To substantiate this statement, in Fig. 3(b) we compare the *y*-averaged Coulomb stress on the plane *z_P_* = 2 *mm* above the slider-bottom surface (*τ_C_*(*x*, *z_P_*), solid green curve) with the corresponding quantity at frictional the interface 
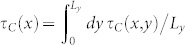
 ( solid magenta curve). As it can be clearly seen, the Coulomb stress value may suffer large fluctuations according to the sample position where it is measured. Although the authors of the experiments in^2^ were careful to perform the measurements at locations *x* to “avoid the effects of large stress gradients”, the agreement shown in Fig. 3 and the analytical calculations in Suppl. Mat. sec. VIII demonstrate that large fluctuations in the (positive) Coulomb stress appear in the bulk of the material, and are mainly due to the internal shear stress gradient resulting from the lateral shear applied. A pictorial intuitive illustration of this argument is reported in Fig. 4, where the *y*-averaged Coulomb stress *τ_C_*(*x*, *z*) is shown for several values of the adiabatic force *F_S_*, see also Movie S2. Regions where *τ_C_*(*x*, *z*) exceeds zero occur even before precursors nucleation (Fig. 4(a)) and even far from the trailing edge. This may lead one to believe incorrectly that the friction coefficient is not a material constant. In the same Fig. 4 (bottom panels) we report the full Coulomb stress *τ_C_*(*x*, *y*) at the plane (*z* = *z_P_*), showing that the average over *y* is indeed a correct protocol to average out the noise-induced fluctuations.

The bulk fluctuations of Coulomb stress are also present while considering a fully tensorial elasticity model. As a matter of fact, in the Suppl. Mat. sec. X we report shear and normal stress calculated by means of a finite element model (FEM). In Fig. S12 we plot *τ_C_*(*x*, *y* = *L_y_*/2, *z*) arising from FEM simulations: large gradients of Coulomb stress make any claim about the local value of *μ* highly questionable. This is seen before any detachment occurred at the interface (panel (a)), and also when a portion of the contact area is disconnected from the rough surface (panels (b)–(d)). We notice that in this case no analytical calculations can be carried out in the fashion of Suppl. Mat. sec. VIII, but the shear and normal stresses are numerically obtained by means of the FEM software. In particular we stress once again that the the normal component of the positive Coulomb stress *σ*(*x*, *y*, *z*) is largely influenced by the shear force *F_S_* as opposite to Eq. S81.

### Role of disorder in front nucleation

The role of the substrate roughness heterogeneity on the nucleation of front appears, from Eqs.(S92),(S94), rather complex. In general one can say that heterogeneity amplifies and modulate the different contributions to normal and shear interface stresses *σ_surf_* and *τ_surf_*. For narrow roughness distributions (in most of the experiments the roughness appears to be a very well-controlled parameter), we do not expect that the substrate disorder may play a major role promoting or suppressing the precursors dynamics, at least not comparable to the role expressed by the force-induced stress gradients. This is what clearly appears from our analysis, in step with the simplified 1D model^10^ and with the experimental outcomes. It is possible, however, that large roughness fluctuations may induce internal stress gradients leading to precursors nucleation, even in regions far from the trailing edge. This is a particularly interesting issue, since the precursor could originate as a stable detachment droplet, irrespective of the type of loading exerted (whether lateral or top shearing). The key question is therefore to determine the conditions for which a detachment droplet constitutes a meta-stable state. The question could be addressed by defining the contact interface energy density as 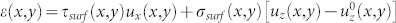
28, and the energy change associated to the transition from an initial stable configuration to a second on which the droplet has formed: 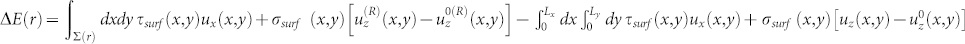, where Σ(*r*) represents the contact surface configuration including the nucleated droplet of average size *r*. A detachment droplet configuration will be stable if the energy penalty Δ*E*(*r*) has a positive maximum for some *r*. Now, asking which physical conditions allow the formation of a stable droplet, means which sample dimensions *L_x_*, *L_y_*, *L_z_*, roughness *w*^2^ and force *F_S_*(< *μF_N_*) give a Δ*E*(*r*) with a positive maximum (with *r* < *L_x_*, *L_y_*, *L_z_*). Unfortunately, due to the intricacy of expressions (S92) and (S94), we could obtain the answer only by numerical simulations. However, albeit one cannot completely exclude that detachment regions appear on length-scales which are well below our and experimental resolution (~1 mm), the set of parameters used in our simulations and in the experiments does not allow for a stable disorder-induced droplet, therefore sliding occurs either by precursors nucleation from the trailing edge or as first-order phase transition for top shearing. On the other hand, it is expected that in thin films (*L_y_*/*L_z_* → ∞, *L_x_*/*L_z_* → ∞), interfacial disorder may induce a droplet nucleation of the kind predicted in Ref. 25. However, since the calculation of Δ*E*(*r*) involves two equilibrium configurations, a quasi-static model is the right candidate to tackle it.

## Discussion

In this paper we have introduced a scalar model for the onset of frictional sliding of a three dimensional elastic object resting on a rough surface. We have devised a scalar elasticity model which allows an analytical treatment of the relevant quantities, and the straightforward implementation of the quasi-static dynamics. This model incorporates, for the first time, mesoscopic laws of contact mechanics at the frictional interface, reproducing with remarkable precision Archard's law and Amonton's first and second laws. Most importantly the scalar model is capable of reproducing with good accuracy the real contact area dynamics, the precursors' envelope dynamics prior to the transition to sliding, and the normal and shear internal stress distributions close to the slider-substrate interface. The model stems from a strong Ansatz, namely that the components of displacements *u_x_* and *u_z_* are decoupled and 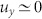. However, the solution of the model is exact: if one accepts the initial Ansatz, one has at hand the analytical expression for any physical observable in static equilibrium. The numerical implementation of the model is required to take into account the statistical heterogeneity inherent to the asperity disorder of the underlying rough substrate.

The first limitation of our model has been discussed previously, and consists in neglecting the Poisson expansion and the sample torque, which can have strong implications only in the case of top uniform shearing conditions, although for very large samples the scalar models conclusions should be respected. Hence no firm general conclusions nor predictions on the occurrence of frictional sliding and precursor dynamics can be drawn based on these observations. Earthquakes faults are mostly driven uniformly from a distance implying that, in average, precursory activity should not be present. Stress gradients and hence precursor activity could, however, arise either due to local heterogeneities or because the fault plane is tilted with respect to the earth crust^29^. The lack of universal scaling forms dictating the precursors evolution, however, makes any forecasting of catastrophic events extremely difficult, especially when we do not know precisely the loading conditions.

The second limitation certainly lies in the quasi-static nature of the model. But what at first glance may seem like a strong simplification is in fact a point of strength. The reasons are the following:

First, the equilibrium problem requires a very limited number of adjustable parameters to set up the model. Our derivation indeed requires the fine tuning of only two parameters, connected to the normal and transverse spring stiffness inspired by the theory of contact mechanics, and whose physical meaning and interpretation are straightforward (see Suppl. Mat. sec. VI). To estimate these parameters, we only need a direct comparison with simple experiments, such as the validation of the Archard's law^30^,^31^ to tune the normal stiffness (see Fig. 6(a)), and an experiment like the one reported in Ref. 32 for the transverse stiffness. This eliminates from the picture a host of dynamical quantities that are often difficult to quantify, or even to justify from the physical point of view. This is the case, for instance, of phenomenolgical friction coefficients interpolating between statics and dynamics employed in 1D^8^ and 2D models^11^,^12^,^13^, or the bulk damping coefficient *γ*, whose numerical value is usually put in by hand^6^,^7^,^8^,^9^,^10^,^11^,^12^,^13^, or of the actual value of the reattachment delay time *τ* responsible for the frictional interface contacts rejuvenation^6^,^7^,^10^. While it is out of doubt that, upon cessation of motion, the contacts at the interface reform and strengthen^5^,^31^, it is very hard to infer the rejuvenation characteristic time *τ* from experimental data. In our model, we did not include the contacts reformation process within our quasi-static protocol, showing that it is not a necessary ingredient to recover the precursors overall profile.

Second, our model may assess which of the observed experimental features are due to the out-of-equilibrium dynamics and which are mostly due to equilibrium properties. For instance, our model is able to recover the precursors steps and the shape of their envelope, but fails to reproduce the increase in the precursors waiting times when the shear is applied at higher and higher *h*. Thus, we can conclude that this intriguing aspect is probably due to inertial effects present when shear is applied through an external spring (see Eq.(22)). To check this experimentally, it would be sufficient to change the spring displacement *U_S_* rate and detect any possible change in the leaps phenomenology. To the contrary, our model allows to establish that the occurrence of precursors is in fact a quasi-static physical process. Any of the equilibrium states reached by the slider during the adiabatic evolution, is just one of the meta-stable configurations in which the system can dwell. This large number of meta-stable states is mainly due to the disorder heterogeneity of the roughness at the interface, and to a much minor degree to the rules adopted to detach the contacts when they satisfy the condition *τ_C_*(*x*, *y*, 0) > 0.

Because of its quasi-static nature, our model cannot reproduce the detachment front dynamics. According to the definition provided in Refs. 1, 2, 4, 27, 33 a detachment front indicates a drastic reduction of the real area of contact which takes place on time scales which are roughly in the millisecond range. The entire precursor experiment occurs instead over a few minutes^4^,^27^. Experiments have revealed three different types of crack-like rupture fronts, slow, sub-Rayleigh, and intersonic (or supershear), according to their propagation velocity through the frictional interface. Precursors advance by arrested front propagation: discrete increments, indeed, occur by rupturing the contact interface at a velocity which corresponds to sub-Rayleigh fronts at the begining, and to slow fronts close to the sliding transition^27^. In particular a final slow front is responsible of the static-to-dynamics frictional sliding. Our model does not capture the crack-like propagation of fronts, since the fronts are a dynamical out-of-equilibrium processes in between two equilibrium states, namely between precursors. Nevertheless, our model might substantiate the experimental observation on the relation between precursors appearance and slow fronts triggering the frictional sliding. As a matter of fact, in Fig. 3 we were able to reproduce quantitatively the shear and normal stress profiles before any precursor nucleation occurred. In Ref. 2 these stress distributions were related to the ensuing slow rupture front (see Fig. 6A in^2^). Thus it is possible to argue that whenever we observe a precursor activity, the transition to sliding is triggered by slow fronts.

To summarize the central finding of our work, three dimensional finite body scalar Green's function makes it possible to investigate the dependence of many physical observables on any sample parameter. Our results show that the evolution of the fronts depends in a non-universal way on the loading conditions and the sample dimensions and shape. Only for some loading condition, the precursors follow a curve that allows for a simple universal rescaling in terms of the sample dimension: this prediction can be experimentally checked. Moreover we have shown that large stress gradients take place not only at the frictional interface but also within the material bulk. These gradients are mainly due to the way the external shear is applied and to the sample geometry, on top of frustrated Poisson expansion and elastic torque. Hence no firm general conclusions nor predictions on the occurrence of frictional sliding and precursor dynamics can be drawn based on these observations.

Earthquakes faults are mostly driven uniformly from a distance implying that, in average, precursory activity should not be present. Stress gradients and hence precursor activity could, however, arise either due to local heterogeneities or because the fault plane is tilted with respect to the earth crust^29^. Measurements of local variations of the Coulomb stress around earthquake faults have been used to assess the correlation between stress accumulation and earthquake triggering^15^,^16^,^17^. Predicting earthquakes based on slip or stress accumulation has been so far an elusive task^18^,^19^, and the reason behind this failure can be addressed in the scenario pictured in our analysis. Indeed, as illustrated, we find that for loading condition leading to large stress gradients, the evolution of the Coulomb stress measured above the contact interface provides only a rough indicator of the ensuing detachment front dynamics, which instead appears to be very well characterized by the real contact area variation. Furthermore, the lack of universal scaling forms dictating the precursors evolution, makes any forecasting of catastrophic events extremely difficult, especially when we do not know precisely the loading conditions.

## Methods

### The scalar model

We consider an elastic PMMA macroscopic body resting in equilibrium on a rough surface. We derive the equations for the displacements of the elastic body subject to external forces, within the scalar elasticity approximation. In particular, we are interested in the solutions for the displacement fields at the frictional interface. There is no direct connection of the model theory to actual elasticity. The latter involves three displacement components and a system of coupled equilibrium equations enforced with boundary conditions.

**Figure 5:**
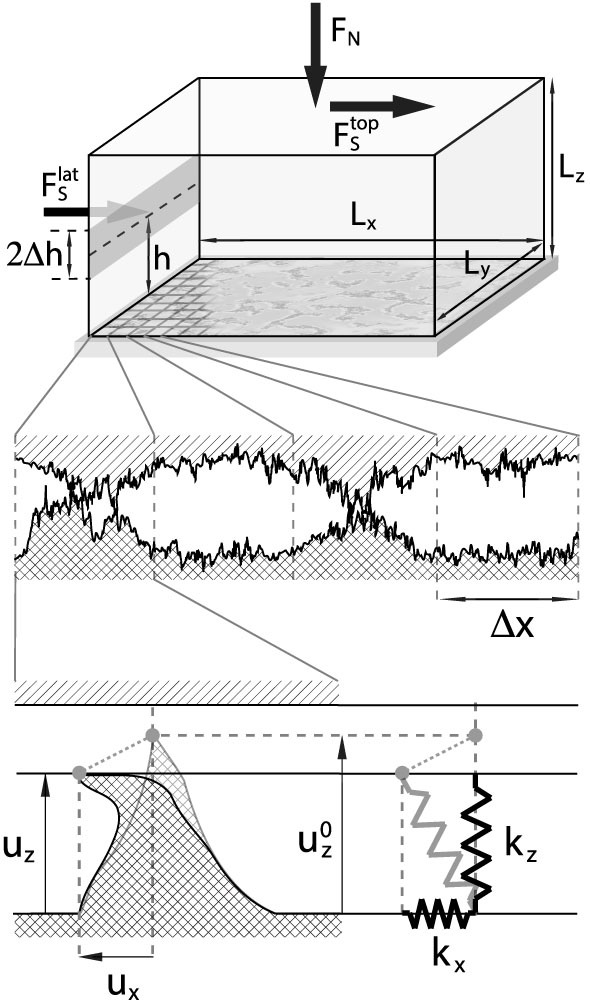
Graphical illustration of the model. We consider a block of dimensions *L_x_*, *L_y_* and *L_z_* in contact with a rough surface (sketched in the middle panel). A normal force *F_N_* and a shear force 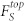 are applied uniformly on the top surface along *z* and *x* respectively, a lateral force 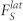 is applied on the sample trailing edge over a rectangular region of width 2 *h* at height *h*. The bottom surface of the block is discretized on a grid of size  *x* ×  *y* (we invariably chose  *y* =  *x* = 1 mm, see also Fig. S1). Each grid element central point may form an elastic contact with the rough surface, that is modelled by a set of elastic asperities of height 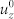, and effective transverse and normal stiffness *k_x_* and *k_z_*, respectively.

Let us first illustrate the physical system that our model aims at reproducing, which coincides with the experimental setup described in Refs. 1,2,3,4, 14, 24, see Fig. 5. The experiments were conducted using two PMMA blocks in contact, one on top of the other. The top block, of dimensions 140,150,200 mm × 6 mm × 75,100 mm (according to the different experiments performed), was pushed against a bottom block of dimensions 250 mm × 30 mm × 28 mm in the 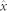, 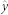 and 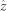 directions respectively. In general, the condition 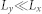, *L_z_* was always satisfied. The two blocks were pushed together by a normal load *F_N_* and, while the bottom block was fixed, the top block was subject to a shearing process by means of the lateral force *F_S_* applied solely on the 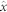 direction. This experimental system was usually adopted, with the only exception of Ref. 2, where the experiments were also conducted clamping the top block at the top edge and applying the shear *F_S_* to the bottom block. However, the relative blocks movement is constrained by the frictional resistance at the interface offered by roughness-induced surface forces. In our model, we consider for simplicity the bottom block to be infinite (*the substrate*) and only the top block shearing. The surface stresses at the interfaces, *φ_surf_*(*x*, *y*, 0), are formally distributions accounting for the spatial hetereogeneity of the PMMA roughness.

Since no external force is acting on the 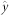 direction, and because the sample geometry fulfills the condition 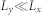, *L_z_*, we have assumed
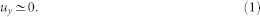
Moreover, the scalar elasticity yields that the stress tensor satisfies *σ_yk_* = 0 (where *k* = *x*, *y*, or *z*). Thus the scalar equations for the decoupled displacement fields take the following form
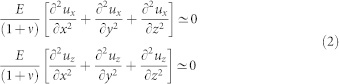
where *E* represents the Young's modulus and *ν* the Poisson's ratio. The scalar elasticity Eqs.(2) can be analyically treatable, once one specifies the proper boundary conditions. At the equilibrium, internal stresses at the surface must counterbalance the external forces acting on the sample. Since we consider a slider of dimensions [*L_x_*, *L_y_*, *L_z_*] the boundary conditions for Eqs.(2) are
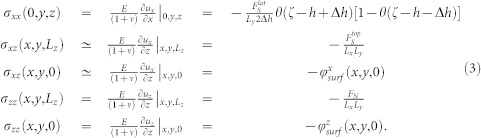
As shown in Fig. 5(a), 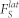 corresponds to a shear force in the *x* direction, applied to the elastic slider on a portion of the plane (0, *y*, *z*) of size *L_y_* × 2Δ*h* centered around *z* = *h* and *θ*(*x*) stands for the Heavyside step function; 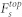 is a shear force (also pointing to the *x* direction) uniformly applied on top of the slider; *F_N_* is the normal force, i.e. a force applied on the entire top plane and pointing toward 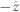; the surface stresses 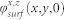 represent the interaction between the elastic body and the rough underlying surface at the plane (*x*, *y*, 0), in the *x* and *z* direction respectively (see Fig. S1). With the boundary conditions (3), we can solve the equilibrium Eqs. (2) for the displacement fields on the slider bottom plane. In technical term, we have to solve two independent Laplace equations with von Neuman boundary conditions. The solutions of Eqs.(2) are obtained by generalizing to three dimensions the corresponding solution for the von Neuman problem in two dimensions^34^. The result reads
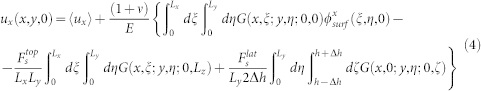

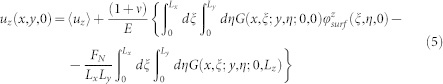
where *G*(*x*, *ξ*; *y*, *η*; *z*, *ζ*) is the Green function:
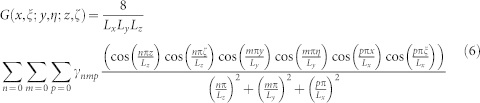
with *γ*_000_ = 0, *γ*_0*mp*_ = *γ_n_*_0*p*_ = *γ_nm_*_0_ = 1/2, *γ*_00*p*_ = *γ*_0*m*0_ = *γ_n_*_00_ = 1/4, and *γ_nmp_* = 1 otherwise. In the Suppl. Mat. sec. I we furnish the analytical procedure for the fast convergence of the sum in Eq.(6).

The quantitites 〈*u_x_*〉 and 〈*u_z_*〉 represent two arbitrary constants corresponding to the displacement fields averaged over the entire sample volume. They must be chosen imposing two additive equilibrium constraints, namely that each component of the the surface forces, over the whole contact surface, must be equal and opposite to the external forces:
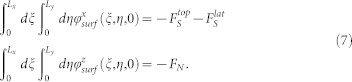
The last step is to provide an adequate analytical expression accounting for the effects of the interface on the slider mechanics, this is done by introducing the surface forces 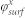 and 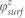. In Suppl. Mat. sec. II we derive the expression of these forces, according to the contact mechanics theories. In first approximation they are both linear in the displacements *u_z_* and *u_x_*, i.e., for the force acting on the 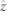 direction, we have
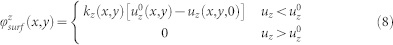
with 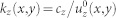; 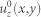 is a random displacement that models the height fluctuations of the rough substrate (see Fig. S1). It is formally an uncorrelated noise extracted from a Gaussian distribution with a variance 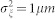, being 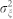 the roughness of the underlying surface^1^,^2^,^3^,^4^,^31^,^35^ (see Suppl. Mat. sec. VI). The interfacial interactions along 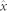 are given by
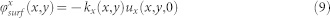
where 
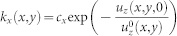
 and 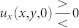. The constant *c_z_* and *c_x_* appearing in the expression of *k_z_* and *k_x_* are the only two adjustable parameters that our model encompasses (see the next subsection and Suppl. Mat. sec. VI). The expressions for *k_x_* and *k_z_* respect the laws of contact mechanics^28^,^36^,^37^ and are entirely motivated by experiments: the transverse (or tangential) stiffness *k_x_* of PMMA is indeed proportional to the normal load^32^, which in general decreases exponentially with the vertical elastic displacement^38^; the normal stiffness is *k_z_* ~ −*dP*/*du_z_*, where 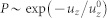 is the squeezing pressure^38^,^39^. Therefore, internal stresses are not decoupled at the interface, as they are connected via the local normal pressure entering the definition of the tangential stiffness *k_x_*.

Finally, introducing the linear expressions (8) and (9) into the Eqs.(5) and (4) respectively, we obtain closed equations for the displacements at the contact plane:
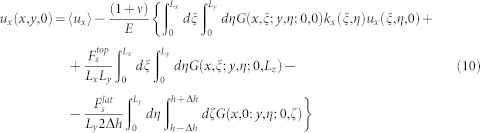

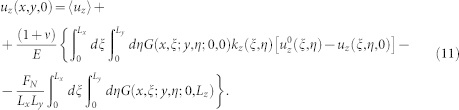


In the former expressions for the interfacial displacements, terms involving the contributions arising from the external shear or normal forces *F_S_* and *F_N_* can be calculated analytically. This is, indeed, one of the novelties that our model introduces: the complete expression of the Green function (see Eq.(6)) allows the determination of any of the force-induced components in the interfcial displacements equations. It will be clear in the next sections that this property entails the critical interpretation of the experimental and numerical results and, in particular, it furnishes precise predictions on the precursors' appeareance and dynamics and their dependence on the slider dimensions. In the Suppl. Mat. sec. III we give the full analytical derivation of the terms proportional to 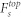, 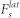 and *F_N_* appearing in Eqs.(10) and (11). Hereby, to simplify the displacements expressions, we introduce the following shorthand notations:
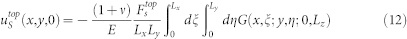

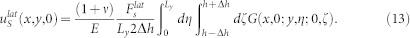

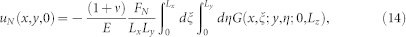
thanks to which the Eqs.(10) and (11) take the form
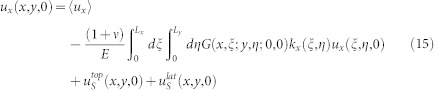
and
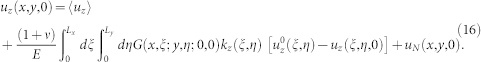


### Discretization, numerical solution and quasi-static dynamics

The slider interfacial displacements, i.e. the solutions of the Eqs. (15) and (16), are achieved by discretization of the slider bottom plane. As a matter of fact, we place a square grid on the contact plane, composed by elements having an area Δ*x* × Δ*y*, so that *L_x_* = *N_x_*Δ*x* and *L_y_* = *N_y_*Δ*y* with Δ*x* = Δ*_y_* = 1 *mm* (see Fig. S1). Albeit the terms in Eqs. (15) and (16) are defined on the entire contact plane (*x*, *y*, 0), we calculate them only on each single point (*x*, *y*), which is the center of the grid element. This is the case, by instance, of the surface forces *φ_x_* and *φ_z_* in Eqs.(9) and (8) respectively, which are formally distributions: we interpret them as acting effectively only on the grid center point, representative of the enclosed area Δ*x* × Δ*y*, as shown in Fig. S1.

In the Suppl. Mat. sec. IV we report the formal derivation of the discretization technique, which leads to the following expressions for the linear inversion Eqs.(15) and (16):
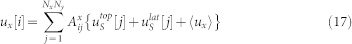
and
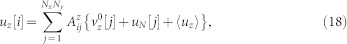
where the matrices 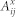 and 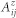 are defined in Eqs.(S44) and (S45) respectively and the vector 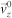 in Eq.(S46). With the former expressions at hand, we can calculate the equilibrium displacements along 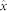 and 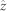, compatible with given values of the external normal and shear forces: we hereby recall that the two constants 〈*u_x_*〉 and 〈*u_z_*〉 are set to ensure that the surface forces counterbalance the external loads (see Eqs.(S48) and (S50)).

In a typical simulation, external shear forces are increased quasi-statically and the actual values of the local interfacial displacements are calculated numerically at the discretized bottom interface thanks to Eqs.(18) and (17) respectively (see Fig. S1). Indeed, we first check for the equilibrium along 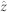, and secondly along 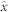. Contact springs are disconnected, i.e. irreversibly broken, whenever the local Coulomb stress satisfies
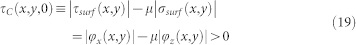
where *μ* represents the static local friction coefficient set to *μ* = 0.5. When the condition (19) is fulfilled, the corresponding interface portion is detached from the underlying surface resulting in a local slip event. Every time a spring is broken, the equilibrium Eqs.(18) and (17) are recalculated with the new boundary condition, i.e. setting to 0 the interfacial forces corresponding to the broken spring.

The overall sliding occurs when none of the interfacial contacts has survived, i.e. when *τ_C_*(*x*, *y*, 0) > 0 across the entire bottom plane (see the flowchart in Fig. S11). This happens when the static friction force is equal to *μF_N_*, satisfying the Amonton's first law. Therefore *μ* represents both the local and global friction coefficient. The detailed protocol of the quasi-static protocol enforced is reported in Suppl. Mat. sec. V.

### Model calibration

**Figure 6:**
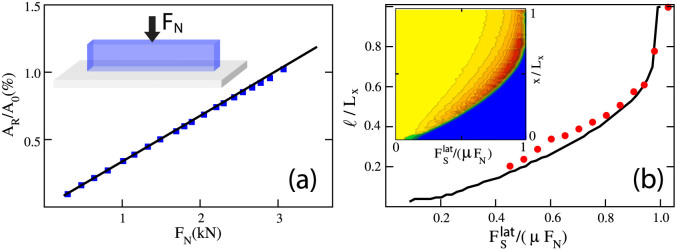
Scalar model numerical calibration. (a) The value of the normal stiffness *k_z_* is set by measuring the change of the real area of contact (in % of the nominal contact area *A*_0_) as a function of the normal load *F_N_*, and comparing the numerical results (squares) with the experimental data from Ref. 31 (solid line). *L_x_* = 30 mm, *L_y_* = 6 mm, *L_z_* = 150 mm. (b) The transverse stiffness *k_x_* is obtained by matching the quasi-static evolution of the precursor position * * (solid black line) with the corresponding experimental data reported in Ref. 4 (red circles). *L_x_* = 140 mm, *L_y_* = 7 mm, *L_z_* = 75 mm, *F_N_* = 3.5 kN. The inset shows the front propagation by imaging the real contact area *A_R_*(*F_S_*) averaged over the *y* direction and normalized to its initial value *A_R_*(0) (the image is the top view of the histogram shown in Fig. 3(b)). Color code: blue reflects a decrease of the real area of contact with respect to the initial value, while red highlights a prominent increase. Precursor fronts correspond to the edge of the blu part of the plot.

**Figure 7:**
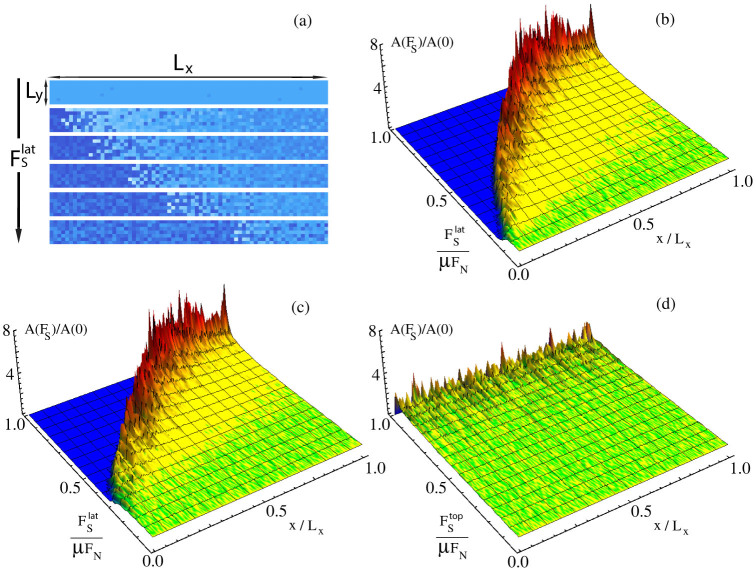
Real area of contact and determination of precursors size. (a) Successive snapshots of the contact area, normalized to its initial value, show the advancement of the slip precursors as 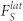 is increased. Dark (pale) blue indicates a decrease (increase) in the contact area. (a)–(c) Quasi-static evolution of the average real area of contact 
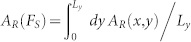
 (normalized to the zero-shear value *A_R_*(0)) for three type of loading conditions: with a rod (*h* = 6 *mm*,  *_h_* = 2 mm) (b), uniformly from a side (c), and uniformly from top (d). *F_N_* = 2.7 kN, *L_x_* = 200 mm, *L_y_* = 7 mm, *L_z_* = 75 mm. Color map goes from blue 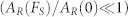 to red 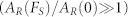: for any value of *F_S_*, blu region corresponds to the precursor size, and the boundary between blu and red/yellow regions represents the precursor size * *. Regions close to the trailing edge experience a decay of the real contact area as *F_S_* is adiabatically increased, whereas the real area of contact area considerably grows on the opposite side ((a) and (b)). When the sample is loaded uniformly from top, the sliding takes place without precursors appearence (c).

One of the key features of our scalar quasi-static model is that Archard's principle is imposed at the mesoscopic scale^30^ since the area of real contact of a grid element is function of the vertical load acting on it, i.e. 
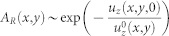
, see Eq.(S61). This is in agreement with the mechanics of the interfacial asperities, whose transverse stiffnesses are 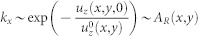, and with the elastic-response picture emerging from the experiment of Berthoud and Baumberger^32^. The definition of the on-site real contact area offers the advantage of controlling the fluctuations of the total contact area during the entire quasi-static evolution. As a matter of fact, it is possible to define the the total real area of contact as
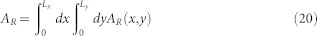
and monitor its change as a function of the applied loads *F_N_* and *F_S_*. This is a notable progress provided by our model when compared with previous 1D^6^,^7^,^8^,^10^,^14^ and 2D models^11^,^13^,^40^, allowing for a direct quantitative comparison with experiments whose main analysis tool is, indeed, the observation of the real contact area evolution^1^,^2^,^4^,^31^. We demonstrate the validity of the Archard's principle at macroscopic scale (see Figs. 6(a)), i.e. *A_R_* increases linearly with the applied vertical force *F_N_*. Moreover, our model provides the remarkable result that *A_R_* only depends on the load *F_N_* and not on the nominal area *L_x_* × *L_y_*, what is commonly known as the Amonton's second law (Fig. S2, S3). As the shear force *F_S_* is adiabatically increased on the other hand, we detect the variation of the real contact area as illustrated in Movie S1 and Fig. 7 for three typical loading conditions used in experiments^1^,^2^,^4^,^31^.

In Suppl. Mat. sec. VI we discuss the calibration of the parameters appearing in our model. The only parameters that have to be adjusted are the two constants *c_x_* and *c_z_* which define the local stiffnesses. To do so, we have to compare the variation of *A_R_* as a function of *F_N_* and *F_S_* provided by our numerical simulations, with the corresponding variations observed in the experiments.

The normal stiffness is obtained by measuring the real contact area as a function of the normal load when no shear is applied, and tuning *c_z_* until the resulting area matches that reported in Ref. 31 (see Fig. 6(a)). Indeed, as detailed in the Suppl. Mat. sec. VI, we define the total real area of contact (20) in the discrete form as
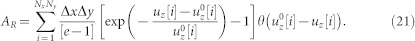
Changing the value of the constant *c_z_* corresponds to change the equilibrium set of *u_z_*[*i*] given by Eq.(18): higher is *c_z_*, stiffer are the interfacial springs, smaller will get the coresponding real contact area. The best value for 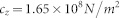 yields the curve reported in Fig. 6(a), showing a remarkable agreement with the experimental observation.

To determine the transverse stiffness, we compare the quasi-static evolution of *A_R_* detected in experiments with the corresponding one obtained from simulations (Fig. 7(a)). In particular, we consider a block of dimensions *L_x_* = 140 mm *L_y_* = 6 mm and *L_z_* = 75 mm under a normal load *F_N_* = 3.3 kN and increase adiabatically the lateral force 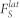 applied at height *h* = 6 mm as in Ref. 4. As shown in Fig. 6(b), as the lateral shear force is increased, the portion of contact area close to the trailing edge decreases drastically. According to the definition furnished in Ref. 4, precursors correspond to the regions of the frictional interface which undergo a reduction of the area of real contact, for values of the applied shear well before the static frictional force. A pictorial view of the adiabatic precursor evolution is reported in the inset of Fig. 6(b), where the color code represents the variation of the average local contact area with respect to its value at *F_S_* = 0. The boundary between the portion of contact surface which has decreased and that which has increased during the shearing process, determines the precursor size *ℓ*. This yieldis a curve that we compare with experiments to estimate the best value of 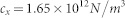 (see the caption of Fig. 6(b) and Suppl. Mat. sec. VI for more details).

### Loading mode and stick-slip events

**Figure 8:**
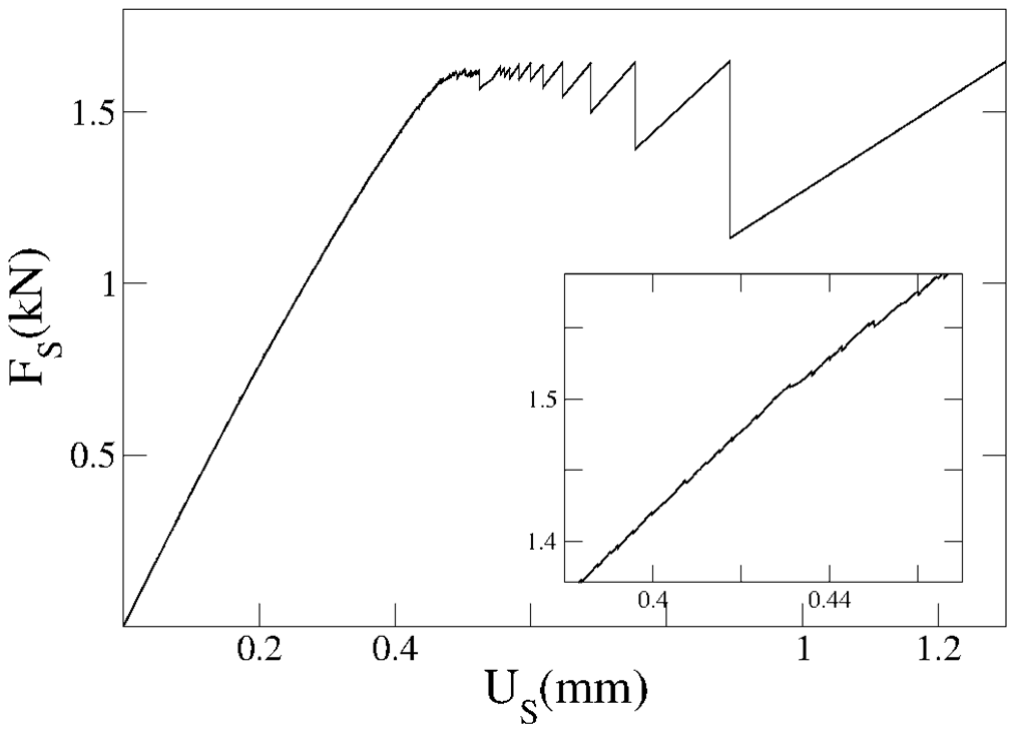
Stick-slip under displacement controlled driving. We report the force on the driving spring as a function of the imposed displacement for the following same sample parameters: *L_x_* = 140 mm, *L_y_* = 7 mm, *L_z_* = 75 mm, *F_N_* = 3.5 kN. A magnification of the curve is reported in the inset showing the precursory stick slip events.

Throughout the paper, we will consider the conceptually simple case in which the sample is loaded by imposing a constant shear force on the appropriate boundaries. This means that we will adopt *F_S_* as the adiabatic variable (quasi-static parameter), and calculate the equilibrium interfacial displacement from Eqs.(17) and (18) each time that *F_S_* is slowly increased. This leads to discrete “leapfroggy” precursors for which we study the continuum envelope as reported for instance in Fig. 6(b). However, the discrete nature of the precursors dynamics is more apparent if we drive the system as in the experiments reported in Ref. 4, where the lateral force is applied through a spring with elastic constant *K_S_* = 4 × 10^6^ N/m. To model this case, we replace the external force appearing in Eq.(4) with the expression
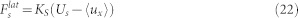
where *U_S_* is the externally applied displacement, which now corresponds to the adiabatic adjustable parameter. 〈*u_x_*〉, on the other hand, has the same meaning as the force-controlled protocol. In Fig. 8, we report the evolution of the spring force (Eq.(22)) as a function of the applied displacement for a typical simulation. Small stick-slip events, corresponding to discrete precursors leaps, are shown in the inset of Fig. 8, closely resembling the experimental observations. Increasing the displacement further leads to larger stick-slip events that in the constant-force case correspond to the last system size spanning event, that leads to the slip of the entire block. More details on the solution of the elastic equations for this particular case can be found in Suppl. Mat. sec. VII.

Our model does not encompasses the rejuvenation of the real area of contact once the precursor has passed, because once a spring is broken no rehealing is allowed. However, in previous models such those in Ref.s 10, 11, 13, 40, once a precursor has detached a portion of interface, the corresponding interfacial contacts always reform, and the whole previous precursor path is broken again by each new precursor. This clearly contradicts the experimental evidence^4^, where no rehealing of the real area of contact can be appreciated between subsequent precursor jumps, and the discrete jumps in the precursors dynamics can be observed only by displaying the derivative 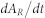 (see by instance Fig. 6(a) of Ref. 4 or Fig.14 of Ref. 27).

## Author Contributions

A.T., A.B. and S.Z. designed the model, A.T. performed analytical calculations, A.B. wrote the code, A.B. and A.T. performed numerical simulations, A.B. and A.T. analyzed the data and prepared the figures, A.B. prepared the movies, A.T. wrote the supplementary text, S.Z. and A.T. wrote the manuscript. S.S. elaborated and performed the simulations on FEM model. All authors discussed the results and implications and commented on the manuscript at all stages.

## Supplementary Material

Supplementary Information Suplementary Information for Scalar model for frictional precursors dynamics

Supplementary Information Movie S1

Supplementary Information Movie S2
