# Supplementary material for: Scalar model for frictional precursors dynamics
Source: Sci Rep. 2015 Feb 2;5:8086. doi: 10.1038/srep08086 (PMC4313121; doi:10.1038/srep08086)
Supplement: Supplementary Information — Suplementary Information for Scalar model for frictional precursors dynamics [file srep08086-s1.pdf]

Supplementary Information for "Scalar model for frictional precursors dynamics"

Alessandro Taloni

CNR-IENI, Via R. Cozzi 53, 20125 Milano, Italy

Andrea Benassi

Empa, Swiss Federal Laboratories for Materials Science and Technology, CH-8600 Dübendorf, Switzerland.

Stefan Sandfeld

Institute of Materials Simulation (WW8), Department of Materials Science, University of Erlangen-Nürnberg (FAU), Dr.-Mack-Str. 77, 90762 Fürth, Germany

Stefano Zapperi

CNR-IENI, Via R. Cozzi 53, 20125 Milano, Italy and ISI Foundation, Via Alassio 11/C 10126 Torino, Italy

Contents

I. Rapidly converging representation of the scalar Green's function 2

II. Surface forces 5

III. External force contributions to the interfacial displacements 7

IV. Model discretization 8

V. Quasi-static dynamics 12

VI. Model calibration 14

VII. Displacement controlled shear 16

VIII. Calculation of Coulomb stress 18

IX. Geometrical dependence of front precursors 21

X. The finite element model 24

A. Model calibration and stress profiles 24

References 25

I. Rapidly converging representation of the scalar Green's function

Albeit in principle Eqs.(4), (5) provide the required displacements on the contact plane for any value of the external forces, in practice they are of very limited usefulness, owing to the extremely poor convergence properties of the Green's function in Eq.(6) [1]. In this section we derive an expression for Eq.(6) which is rapidly converging. Firstly, we expand it by assigning to the coefficients γ_{mnp} their explicit values:

$$
\begin{aligned}
G(x,\xi;y,\eta;z,\zeta) = \tfrac{8}{L_x L_y L_z} \Bigg\{ & \tfrac{1}{4}\sum_{p=1} \frac{\cos\left(\frac{p\pi x}{L_x}\right)\cos\left(\frac{p\pi\xi}{L_x}\right)}{\left(\frac{p\pi}{L_x}\right)^2} + \\
& + \tfrac{1}{4}\sum_{m=1} \cos\left(\frac{m\pi y}{L_y}\right)\cos\left(\frac{m\pi\eta}{L_y}\right)\left[\frac{1}{\left(\frac{m\pi}{L_y}\right)^2} + 2\sum_{p=1}\frac{\cos\left(\frac{p\pi x}{L_x}\right)\cos\left(\frac{p\pi\xi}{L_x}\right)}{\left(\frac{m\pi}{L_y}\right)^2+\left(\frac{p\pi}{L_x}\right)^2}\right] + \\
& + \tfrac{1}{4}\sum_{n=1} \cos\left(\frac{n\pi z}{L_z}\right)\cos\left(\frac{n\pi\zeta}{L_z}\right)\left[\frac{1}{\left(\frac{n\pi}{L_z}\right)^2} + 2\sum_{m=1}\frac{\cos\left(\frac{m\pi y}{L_y}\right)\cos\left(\frac{m\pi\eta}{L_y}\right)}{\left(\frac{n\pi}{L_z}\right)^2+\left(\frac{m\pi}{L_y}\right)^2}\right] + \\
& + \tfrac{1}{2}\sum_{p=1}\sum_{n=1} \cos\left(\frac{p\pi x}{L_x}\right)\cos\left(\frac{p\pi\xi}{L_x}\right)\cos\left(\frac{n\pi z}{L_z}\right)\cos\left(\frac{n\pi\zeta}{L_z}\right) \times \\
& \times \left[\frac{1}{\left(\frac{n\pi}{L_z}\right)^2+\left(\frac{p\pi}{L_x}\right)^2} + 2\sum_{m=1}\frac{\cos\left(\frac{m\pi y}{L_y}\right)\cos\left(\frac{m\pi\eta}{L_y}\right)}{\left(\frac{m\pi}{L_y}\right)^2+\left(\frac{n\pi}{L_z}\right)^2+\left(\frac{p\pi}{L_x}\right)^2}\right]\Bigg\}.
\end{aligned}
\tag{S1}
$$

We can then study the different terms appearing in the former expression. The first sum is $\frac{1}{4}\sum_{p=1}\frac{\cos\left(\frac{p\pi x}{L_x}\right)\cos\left(\frac{p\pi\xi}{L_x}\right)}{\left(\frac{p\pi}{L_x}\right)^2} = \mathscr{F}(x,\xi;L_x)$, which can be straightforwardly evaluated [2]

$$
\mathscr{F}(x,\xi;L_x) = \frac{1}{48}\begin{cases} 3\left(x^2+\xi^2\right)+2L_x^2-6\xi L_x & 0 \le x \le \xi \\ 3\left(x^2+\xi^2\right)+2L_x^2-6xL_x & \xi \le x \le L_x. \end{cases}
\tag{S2}
$$

The second term can be recast as [2]

$$
\begin{aligned}
& \tfrac{1}{4}\sum_{m=1} \cos\left(\frac{m\pi y}{L_y}\right)\cos\left(\frac{m\pi\eta}{L_y}\right)\left[\frac{1}{\left(\frac{m\pi}{L_y}\right)^2} + 2\sum_{p=1}\frac{\cos\left(\frac{p\pi x}{L_x}\right)\cos\left(\frac{p\pi\xi}{L_x}\right)}{\left(\frac{m\pi}{L_y}\right)^2+\left(\frac{p\pi}{L_x}\right)^2}\right] = \\
& \tfrac{L_x L_y}{16\pi}\sum_{m=1}\frac{\cos\left(\frac{m\pi|y-\eta|}{L_y}\right)+\cos\left(\frac{m\pi(y+\eta)}{L_y}\right)}{m}\left\{\frac{\cosh\left[\frac{m\pi(L_x-|x-\xi|)}{L_y}\right]}{\sinh\left[\frac{m\pi L_x}{L_y}\right]} + \frac{\cosh\left[\frac{m\pi(L_x-x-\xi)}{L_y}\right]}{\sinh\left[\frac{m\pi L_x}{L_y}\right]}\right\},
\end{aligned}
\tag{S3}
$$

and we can get a compact representation introducing the function

$$
\mathscr{L}(a,b;L_a,L_b) = \frac{L_a L_b}{16\pi}\sum_{m=1}\frac{\cos\left(\frac{m\pi b}{L_b}\right)}{m}\frac{\cosh\left[\frac{m\pi(L_a-a)}{L_b}\right]}{\sinh\left[\frac{m\pi L_a}{L_b}\right]}.
\tag{S4}
$$

Hence Eq.(S3) acquires the final form

$$
\begin{aligned}
& \tfrac{1}{4}\sum_{m=1} \cos\left(\frac{m\pi y}{L_y}\right)\cos\left(\frac{m\pi\eta}{L_y}\right)\left[\frac{1}{\left(\frac{m\pi}{L_y}\right)^2} + 2\sum_{p=1}\frac{\cos\left(\frac{p\pi x}{L_x}\right)\cos\left(\frac{p\pi\xi}{L_x}\right)}{\left(\frac{m\pi}{L_y}\right)^2+\left(\frac{p\pi}{L_x}\right)^2}\right] = \\
& \mathscr{L}\left(|x-\xi|,|y-\eta|;L_x,L_y\right) + \mathscr{L}\left(x+\xi,|y-\eta|;L_x,L_y\right) + \\
& + \mathscr{L}\left(|x-\xi|,y+\eta;L_x,L_y\right) + \mathscr{L}\left(x+\xi,y+\eta;L_x,L_y\right).
\end{aligned}
\tag{S5}
$$

The same can be done for the third term in the righthand side of Eq.(S1).

We now address the last term, that we partially sum as [2]

$$\begin{aligned}
&\sum_{p=1}\sum_{n=1}\frac{\cos\left(\frac{p\pi x}{L_x}\right)\cos\left(\frac{p\pi\xi}{L_x}\right)\cos\left(\frac{n\pi z}{L_z}\right)\cos\left(\frac{n\pi\zeta}{L_z}\right)}{2}\times\\
&\times\left[\frac{1}{\left(\frac{n\pi}{L_z}\right)^2+\left(\frac{p\pi}{L_x}\right)^2}+2\sum_{m=1}\frac{\cos\left(\frac{m\pi y}{L_y}\right)\cos\left(\frac{m\pi\eta}{L_y}\right)}{\left(\frac{m\pi}{L_y}\right)^2+\left(\frac{n\pi}{L_z}\right)^2+\left(\frac{p\pi}{L_x}\right)^2}\right]=\\
&\frac{L_y}{8\pi}\sum_{p=1}\left[\cos\left(\frac{p\pi|x-\xi|}{L_x}\right)+\cos\left(\frac{p\pi(x+\xi)}{L_x}\right)\right]\sum_{n=1}\frac{\cos\left(\frac{n\pi|z-\zeta|}{L_z}\right)+\cos\left(\frac{n\pi(z+\zeta)}{L_z}\right)}{\sqrt{\left(\frac{p}{L_x}\right)^2+\left(\frac{n}{L_z}\right)^2}}\times\\
&\times\left\{\frac{\cosh\left[\pi\sqrt{\left(\frac{p}{L_x}\right)^2+\left(\frac{n}{L_z}\right)^2}(L_y-|y-\eta|)\right]+\cosh\left[\pi\sqrt{\left(\frac{p}{L_x}\right)^2+\left(\frac{n}{L_z}\right)^2}(L_y-y-\eta)\right]}{\sinh\left[\pi\sqrt{\left(\frac{p}{L_x}\right)^2+\left(\frac{n}{L_z}\right)^2}L_y\right]}\right\}.
\end{aligned} \tag{S6}$$

Defining the function

$$\begin{aligned}
&\mathscr{H}\left(a,b,c;L_a,L_b,L_c\right)=\\
&\frac{L_b}{8\pi}\sum_{p=1}\sum_{n=1}\frac{\cos\left(\frac{p\pi a}{L_a}\right)\cos\left(\frac{n\pi c}{L_c}\right)\cosh\left[\pi\sqrt{\left(\frac{p}{L_a}\right)^2+\left(\frac{n}{L_c}\right)^2}(L_b-b)\right]}{\sqrt{\left(\frac{p}{L_a}\right)^2+\left(\frac{n}{L_c}\right)^2}\sinh\left[\pi\sqrt{\left(\frac{p}{L_a}\right)^2+\left(\frac{n}{L_c}\right)^2}L_b\right]}
\end{aligned} \tag{S7}$$

we express Eq.(S6) as

$$\begin{aligned}
&\sum_{p=1}\sum_{n=1}\frac{\cos\left(\frac{p\pi x}{L_x}\right)\cos\left(\frac{p\pi\xi}{L_x}\right)\cos\left(\frac{n\pi z}{L_z}\right)\cos\left(\frac{n\pi\zeta}{L_z}\right)}{2}\times\\
&\times\left[\frac{1}{\left(\frac{n\pi}{L_z}\right)^2+\left(\frac{p\pi}{L_x}\right)^2}+2\sum_{m=1}\frac{\cos\left(\frac{m\pi y}{L_y}\right)\cos\left(\frac{m\pi\eta}{L_y}\right)}{\left(\frac{m\pi}{L_y}\right)^2+\left(\frac{n\pi}{L_z}\right)^2+\left(\frac{p\pi}{L_x}\right)^2}\right]=\\
&\mathscr{H}\left(|x-\xi|,|y-\eta|,|z-\zeta|;L_x,L_y,L_z\right)+\\
&+\mathscr{H}\left(x+\xi,|y-\eta|,|z-\zeta|;L_x,L_y,L_z\right)+\\
&+\mathscr{H}\left(|x-\xi|,y+\eta,|z-\zeta|;L_x,L_y,L_z\right)+\\
&+\mathscr{H}\left(|x-\xi|,|y-\eta|,z+\zeta;L_x,L_y,L_z\right)+\\
&+\mathscr{H}\left(x+\xi,y+\eta,|z-\zeta|;L_x,L_y,L_z\right)+\\
&+\mathscr{H}\left(x+\xi,|y-\eta|,z+\zeta;L_x,L_y,L_z\right)+\\
&+\mathscr{H}\left(|x-\xi|,y+\eta,z+\zeta;L_x,L_y,L_z\right)+\mathscr{H}\left(x+\xi,y+\eta,z+\zeta;L_x,L_y,L_z\right).
\end{aligned} \tag{S8}$$

By inserting the expressions (S2), (S5) and (S8) in (S1) we obtain the desidered result. However a close analysis reveals that the ensuing formula does provide a fast convergence for any set of points $(x,\xi;y,\eta;z,\zeta)$, but for those who lie on the hypersurface $x\neq\xi, y=\eta, z=\zeta$. In this case the Green function can be recast in the following form:

$$
\begin{aligned}
G(x,\xi;y,\eta;z,\zeta) = \tfrac{8}{L_x L_y L_z} \{&\mathscr{F}(y,\eta;L_y)+\\
&\mathscr{L}(|y-\eta|,|x-\xi|;L_y,L_x)+\mathscr{L}(|y-\eta|,x+\xi;L_y,L_x)+\\
&+\mathscr{L}(y+\eta,|x-\xi|;L_y,L_x)+\mathscr{L}(y+\eta,x+\xi;L_y,L_x)+\\
&+\mathscr{L}(|x-\xi|,|z-\zeta|;L_x,L_z)+\mathscr{L}(x+\xi,|z-\zeta|;L_x,L_z)+\\
&+\mathscr{L}(|x-\xi|,z+\zeta;L_x,L_z)+\mathscr{L}(x+\xi,z+\zeta;L_x,L_z)+\\
&+\mathscr{H}(|y-\eta|,|x-\xi|,|z-\zeta|;L_y,L_x,L_z)+\\
&+\mathscr{H}(|y-\eta|,x+\xi,|z-\zeta|;L_y,L_x,L_z)+\\
&+\mathscr{H}(y+\eta,|x-\xi|,|z-\zeta|;L_y,L_x,L_z)+\\
&+\mathscr{H}(|y-\eta|,|x-\xi|,z+\zeta;L_y,L_x,L_z)+\\
&+\mathscr{H}(y+\eta,x+\xi,|z-\zeta|;L_y,L_x,L_z)+\\
&+\mathscr{H}(|y-\eta|,x+\xi,z+\zeta;L_y,L_x,L_z)+\\
&+\mathscr{H}(y+\eta,|x-\xi|,z+\zeta;L_y,L_x,L_z)+\mathscr{H}(y+\eta,x+\xi,z+\zeta;L_y,L_x,L_z)\}\,.
\end{aligned}
\tag{S9}
$$

II. Surface forces

To complete the solutions in Eqs.(4), (5) for the displacements fields at the contact plane, we have to introduce a functional form for the surface stresses $\varphi^{x,z}_{surf}$. This expression represents the slider-surface interaction, embodying the microsopic details into a coarse-grained description. Albeit the surface forces $\varphi^{x,z}_{surf}(x,y)$ are formally distributions defined on the contact plane $(x,y,0)$, they must be interpreted as effectively acting only on a single point (x,y) representative of the surface portion $\Delta x \times \Delta y$ that surrounds it (Fig.S1). In the next section we will provide a clear description of the slider-surface interface discretization scheme. Here we only clarify the assumptions which $\varphi^{x,z}_{surf}(x,y)$ are built from:

- the surface element $\Delta x \times \Delta y$ behaves as a macroscopic object, fulfilling the macroscopic laws of friction;
- the surface forces are purely elastic, i.e. linear in the displacement fields.

We consider first the surface force along the z direction φ^{z}_{surf}. Experimental evidence [3]shows that an elastic body, under a squeezing pressure P, exhibits an average surface separation $\langle u_z \rangle$ which decreases with P as

$$P \sim e^{-\frac{\langle u_z \rangle}{u_z^0}}; \tag{S10}$$

here u_z^0 depends on the nature of the surface rougheness but is independent on P. Thus, the first assumption requires that the internal stress $\sigma_{zz}(x, y, 0)$ on the microscopical surface $\Delta x \times \Delta y$ centered around (x, y), can be written as

$$\sigma_{zz}(x, y, 0) \sim -e^{-\frac{u_z(x,y,0)}{u_z^0(x,y)}}. \tag{S11}$$

The minus sign relates to the tensorial nature of $\sigma_{zz}(x, y, 0)$, always pointing along the $-\hat{z}$ direction. Now, since at the equilibrium $\sigma_{zz}(x, y, 0) = -\varphi^z_{surf}(x, y)$, as noticed in Ref. [4] the local interfacial stiffness is given by

$$k_z(x, y) = -\frac{d\varphi^z_{surf}(x, y)}{du_z(x, y, 0)} = \frac{\varphi^z_{surf}(x, y)}{u_z^0(x, y)} \sim \frac{e^{-\frac{u_z(x,y,0)}{u_z^0(x,y)}}}{u_z^0(x, y)}, \tag{S12}$$

which, for $u_z(x, y, 0) \simeq u_z^0(x, y)$ can be recast as

$$k_z(x, y) = \frac{c_z}{u_z^0(x, y)}, \tag{S13}$$

where c_z is a constant to be set. Thus, the surface stress can be written in the following linear form

$$\varphi^z_{surf}(x, y) = k_z(x, y) \left[u_z^0(x, y) - u_z(x, y, 0)\right] = \frac{c_z}{u_z^0(x, y)} \left[u_z^0(x, y) - u_z(x, y, 0)\right] \tag{S14}$$

fulfilling the second assumption. We notice that the previous form holds only for $u_z^0(x, y) > u_z(x, y, 0)$, and $\varphi^z_{surf}(x, y) = 0$ when $u_z^0(x, y) < u_z(x, y, 0)$ $(u_z(x, y, 0) \geq 0)$, see Fig.1(a). We now consider the interfacial stress along x $\varphi^x_{surf}(x, y)$. For small displacements $u_x(x, y, 0)$, the second assumption requires that it can be expanded to first order as

$$\varphi^x_{surf}(x, y) = -k_x(x, y)u_x(x, y, 0) \tag{S15}$$

where $u_x(x, y, 0) \gtreqless 0$. We have to find an expression for $k_x(x, y)$. From Eq.(S11) the normal load acting on the surface element $\Delta x \Delta y$ is given by

$$F_N(x, y) = \Delta x \Delta y \left|\sigma_{zz}(x, y, 0)\right| \sim \Delta x \Delta y e^{-\frac{u_z(x,y,0)}{u_z^0(x,y)}}. \tag{S16}$$

Now, the experiment in [5] shows that an elastic body behaves like an harmonic spring (with constant K_x), if subject to a small shear force: the spring constant is linear in the applied load, i.e.

$$K_x(F_N) \sim F_N. \tag{S17}$$

Hence, fulfilling the first hypothesis, we can assume that the surface element $\Delta x \times \Delta y$ is characterized by a microscopic transverse spring stiffness

$$k_x(x,y) = c_x e^{-\frac{u_z(x,y,0)}{u_z^0(x,y)}}, \tag{S18}$$

with c_x constant to be set. Thanks to (S15) and (S18) we can finally write the interfacial stress along the x direction as

$$\varphi^x_{surf}(x,y) = -k_x(x,y)u_x(x,y,0) = -c_x e^{-\frac{u_z(x,y,0)}{u_z^0(x,y)}} u_x(x,y,0). \tag{S19}$$

Finally, introducing the linear expressions Eqs.(S19) and (S14) in Eqs.(4) and (5) respectively, we obtain the closed Eqs. (10), (11).

III. External force contributions to the interfacial displacements

In the expressions (10) and (11) for the interfacial displacements, terms involving the contributions arising from the external shear or normal forces F_S and F_N can be calculted analytically.

In Eq.(10) we can evaluate the term proportional to the shear force applied uniformly at the top surface F_s^{top}. According to [2], plugging Eq.(6) in Eq.(4), we obtain

$$\frac{F_s^{top}}{L_x L_y}\int_0^{L_x} d\xi \int_0^{L_y} d\eta\, G(x,\xi;y,\eta;0,L_z) = -\frac{L_z}{6L_xL_y}F_s^{top}. \tag{S20}$$

Hence, the contribution arising from applied shear on the top surface is constant for any point (x,y) on the contact surface. We can thus define a component of the displacement at the interface

$$u_S^{top}(x,y,0) = -\frac{(1+\nu)}{E}\frac{F_s^{top}}{L_xL_y}\int_0^{L_x} d\xi \int_0^{L_y} d\eta\, G(x,\xi;y,\eta;0,L_z) \tag{S21}$$

which does not display any dependence on (x, y). On the other hand, the contribution due to the lateral shear F_s^{lat} can be evaluated as

$$\begin{aligned}&\tfrac{F_s^{lat}}{L_y 2\Delta h}\int_0^{L_y} d\eta \int_{h-\Delta h}^{h+\Delta h} d\zeta\, G(x,0;y,\eta;0,\zeta) = \tfrac{L_x}{L_y L_z} F_s^{lat}\left[\tfrac{x^2}{2L_x^2} - \tfrac{x}{L_x} + \tfrac{1}{3}\right] + \\ &+ \tfrac{F_s^{lat} L_z}{\pi^2 L_x L_y \Delta h}\left\{\tfrac{(h+\Delta h)\pi^2}{12}\left[\tfrac{(h+\Delta h)^2}{L_z^2} - 3\tfrac{(h+\Delta h)}{L_z} + 2\right] - \right.\\ &- \tfrac{(h-\Delta h)\pi^2}{12}\left[\tfrac{(h-\Delta h)^2}{L_z^2} - 3\tfrac{(h-\Delta h)}{L_z} + 2\right] + \\ &\left. + L_x \sum_{n=1} \tfrac{\cosh\left[n\pi\frac{(L_x - x)}{L_z}\right]}{n^2 \sinh\left[n\pi\frac{L_x}{L_z}\right]}\left[\sin\left(n\pi\tfrac{(h+\Delta h)}{L_z}\right) - \sin\left(n\pi\tfrac{(h-\Delta h)}{L_z}\right)\right]\right\}.\end{aligned} \tag{S22}$$

In the case of a shear force applied uniformly on the slider trailing edge ($x = 0$, $h = \Delta h = \frac{L_z}{2}$), the former expression simplifies to

$$\frac{F_s^{lat}}{L_y L_z}\int_0^{L_y} d\eta \int_0^{L_z} d\zeta\, G(x,0;y,\eta;0,\zeta) = \frac{L_x}{L_y L_z} F_s^{lat}\left[\frac{x^2}{2L_x^2} - \frac{x}{L_x} + \frac{1}{3}\right]. \tag{S23}$$

Again we can define the contribution to the local displacement due to a lateral shear force as

$$u_S^{lat}(x,y,0) = \frac{(1+\nu)}{E}\frac{F_s^{lat}}{L_y 2\Delta h}\int_0^{L_y} d\eta \int_{h-\Delta h}^{h+\Delta h} d\zeta\, G(x,0;y,\eta;0,\zeta). \tag{S24}$$

We can perform the same analysis for normal displacements, obtaining again a constant contribution due to the normal force

$$u_N(x,y,0) = -\frac{(1+\nu)}{E}\frac{F_N}{L_x L_y}\int_0^{L_x} d\xi \int_0^{L_y} d\eta\, G(x,\xi;y,\eta;0,L_z) = \frac{(1+\nu)}{E}\frac{L_z}{6L_x L_y}F_N. \tag{S25}$$

IV. Model discretization

We hereby show the procedure to discretize the contact surface between the slider and the underlying interface. Therefore we take a grid of the slider bottom plane, with an individual element having an area $\Delta x \times \Delta y$, i.e.

$$\begin{aligned} L_x &= N_x \Delta x \\ L_y &= N_y \Delta y \end{aligned} \tag{S26}$$

as it is shown in Fig.S1. The central point (x, y) of each grid element takes the following discrete form

$$
\begin{aligned}
x &= \left(n_x - \tfrac{1}{2}\right)\Delta x \quad n_x \in [1, N_x] \\
y &= \left(n_y - \tfrac{1}{2}\right)\Delta y \quad n_x \in [1, N_y].
\end{aligned} \tag{S27}
$$

The solutions Eqs.10 and 11 at any point (x, y) require the integration over the whole surface, i.e. the sum over the discrete set of the points (ξ, η)

$$
\begin{aligned}
\xi &= \left(n_\xi - \tfrac{1}{2}\right)\Delta x \quad n_\xi \in [1, N_x] \\
\eta &= \left(n_\eta - \tfrac{1}{2}\right)\Delta y \quad n_\eta \in [1, N_y].
\end{aligned} \tag{S28}
$$

Therefore any grid center point (x, y), as well as any point (ξ, η) can be represented by a single index i and j respectively,

$$
\begin{aligned}
i &= (n_y - 1)N_x + n_x = \left(\tfrac{y}{\Delta y} - \tfrac{1}{2}\right)\tfrac{L_x}{\Delta x} + \left(\tfrac{x}{\Delta x} + \tfrac{1}{2}\right) \quad i \in [1, N_x N_y] \\
j &= (n_\eta - 1)N_x + n_\xi = \left(\tfrac{\eta}{\Delta y} - \tfrac{1}{2}\right)\tfrac{L_x}{\Delta x} + \left(\tfrac{\xi}{\Delta x} + \tfrac{1}{2}\right) \quad j \in [1, N_x N_y],
\end{aligned} \tag{S29}
$$

and displacements and surface forces become to one-dimensional vectors made of $N_x N_y$ elements each:

$$
\begin{aligned}
u_z(x, y, 0) &\rightarrow u_z[i] \in \vec{u}_z \\
u_x(x, y, 0) &\rightarrow u_x[i] \in \vec{u}_x \\
u_z^0(x, y) &\rightarrow u_z^0[i] \in \vec{u}_z^0 \\
k_z(x, y) &\rightarrow k_z[i] = \tfrac{c_z}{u_z^0[i]} \in \vec{k}_z \\
k_x(x, y) &\rightarrow k_x[i] = c_x e^{-\frac{u_z[i]}{u_z^0[i]}} \in \vec{k}_x.
\end{aligned} \tag{S30}
$$

Correspondingly the Green's function on the contact plane $(z = \zeta = 0)$ is a $N_x N_y \times N_x N_y$ matrix:

$$
G(x, \xi; y, \eta; 0, 0) \rightarrow G_{ij} \in \hat{G} \tag{S31}
$$

where each element can be calculated analitically thanks to Eqs.(S1)-(S9), once $(x, y; \xi, \eta) \rightarrow (i, j)$ by means of Eq.(S29). It remains to discretize the contributions given by the external foces F_S and F_N to Eq.(10) and Eq.(11). From Eqs.(S20) and (S21), it turns out that the term proportional to F_S^{top} is constant across the entire contact interface, resulting in a constant vector whose components are

$$
u_S^{top}(x, y, 0) \rightarrow u_S^{top}[i] \in \vec{u}_S^{top}. \tag{S32}
$$

On the other hand, the contribution coming from F_S^{lat} always displays a non-trivial x-dependence, be the force applied with a shearing rod (Eq.(S22)) or uniformly on the sample side surface (Eq.(S23)). After discretization of the contact plane, this yields the lateral shear contribution to the $i-$th component of $\vec{u}_x$ which arises from the discretization of the corresponding continuum component (Eq.(S24)):

$$u_S^{lat}(x, y, 0) \;\rightarrow\; u_S^{lat}[i] \in \vec{u}_S^{lat}. \tag{S33}$$

Finally, from Eq.(S25) we find that the contribution given to the displacement u_z by the loading force F_N is constant over the whole interface, so that

$$u_N(x, y, 0) \;\rightarrow\; u_N[i] \in \vec{u}_N. \tag{S34}$$

We can now write the expressions (10) and (11) for the discrete component i of $\vec{u}_x$ and $\vec{u}_z$

$$u_x[i] = -\frac{(1+\nu)}{E} \sum_{j=1}^{N_x N_y} \Delta x \Delta y G_{ij} k_x[j] u_x[j] + u_S^{top}[i] + u_S^{lat}[i] + \langle u_x \rangle \tag{S35}$$

$$u_z[i] = \frac{(1+\nu)}{E} \sum_{j=1}^{N_x N_y} \Delta x \Delta y G_{ij} k_z[j] \left\{ u_z^0[j] - u_z[j] \right\} + u_N[i] + \langle u_z \rangle \tag{S36}$$

where we made use of Eqs.(S14) and (S19). Now, let us introduce the matrices $\hat{k}^x$ and $\hat{k}^z$, defined as

$$k_{ij}^x = \begin{cases} \Delta x \Delta y k_x[j] & i = j \\ 0 & otherwise \end{cases} \tag{S37}$$

$$k_{ij}^z = \begin{cases} \Delta x \Delta y k_z[j] & i = j \\ 0 & otherwise. \end{cases} \tag{S38}$$

Thus expressions (S14) and (S19) can be cast in vectorial form as

$$\vec{u}_x = -\frac{(1+\nu)}{E} \hat{G} \hat{k}^x \vec{u}_x + \vec{u}_S^{top} + \vec{u}_S^{lat} + \langle \vec{u_x} \rangle \tag{S39}$$

$$\vec{u}_z = \frac{(1+\nu)}{E} \left\{ \hat{G} \hat{k}^z \vec{u}_z^0 - \hat{G} \hat{k}^z \vec{u}_z \right\} + \vec{u}_N + \langle \vec{u_z} \rangle, \tag{S40}$$

where we introduce the vectors $\langle \vec{u_x} \rangle$ and $\langle \vec{u_z} \rangle$ whose components are constant. Then we can obtain the solutions by inversion:

$$\vec{u}_x = \left[\hat{I} + \frac{(1+\nu)}{E} \hat{G}\hat{k}^x \right]^{-1} \left\{ \vec{u}_S^{top} + \vec{u}_S^{lat} + \langle \vec{u_x} \rangle \right\} \tag{S41}$$

$$\vec{u}_z = \left[\hat{I} + \frac{(1+\nu)}{E} \hat{G}\hat{k}^z \right]^{-1} \left\{ \frac{E}{(1+\nu)} \hat{G}\hat{k}^z \vec{u}_z^0 + \vec{u}_N + \langle \vec{u_z} \rangle \right\}, \tag{S42}$$

where $\hat{I}$ is the identity matrix. Finally we have to impose the constraints in Eq.(7) that can now be written as

$$\begin{aligned} &\sum_{i=1}^{N_x N_y} \Delta x \Delta y k_x[i] u_x[i] = F_S^{top} + F_S^{lat} \\ &\sum_{i=1}^{N_x N_y} \Delta x \Delta y k_z[i] \left\{ u_z^0[i] - u_z[i] \right\} = -F_N. \end{aligned} \tag{S43}$$

In order to fulfill Eq.(S43) we need the solution of Eqs.(S39) and (S40) for the i-th component of the displacement fields. Introducing the simplified expressions

$$\hat{A}^x = \left[\hat{I} + \frac{(1+\nu)}{E} \hat{G}\hat{k}^x \right]^{-1}, \tag{S44}$$

$$\hat{A}^z = \left[\hat{I} + \frac{(1+\nu)}{E} \hat{G}\hat{k}^z \right]^{-1} \tag{S45}$$

and

$$\frac{E}{(1+\nu)} \hat{G}\hat{k}^z \vec{u}_z^0 = \vec{v}_z^0, \tag{S46}$$

we obtain the final expressions (17), (18). Inserting Eqs.(17) and (18) in Eq.(S43) and making use of Eq.(S37), the x constraint can be written as

$$\langle u_x \rangle \sum_{i=1}^{N_x N_y} \sum_{j=1}^{N_x N_y} A_{ij}^x k_{ii}^x = F_S^{top} + F_S^{lat} - \sum_{l=1}^{N_x N_y} \sum_{i=1}^{N_x N_y} \sum_{j=1}^{N_x N_y} k_{li}^x A_{ij}^x \left\{ u_S^{top}[j] + u_S^{lat}[j] \right\}, \tag{S47}$$

from which

$$\langle u_x \rangle = \frac{F_S^{top} + F_S^{lat} - \sum_{l=1}^{N_x N_y} \sum_{i=1}^{N_x N_y} \sum_{j=1}^{N_x N_y} k_{li}^x A_{ij}^x \left\{ u_S^{top}[j] + u_S^{lat}[j] \right\}}{\sum_{i=1}^{N_x N_y} \sum_{j=1}^{N_x N_y} A_{ij}^x k_{ii}^x}. \tag{S48}$$

In the same way we can handle the constraint along z: from Eq.(S43) we have

$$\begin{aligned}
&\langle u_z\rangle \sum_{i=1}^{N_xN_y}\sum_{j=1}^{N_xN_y} A_{ij}^x k_{ii}^z = \\
&F_N + \sum_{i=1}^{N_xN_y}\sum_{j=1}^{N_xN_y} k_{ij}^z[i]u_z^0[j] - \sum_{i=1}^{N_xN_y}\sum_{j=1}^{N_xN_y}\sum_{l=1}^{N_xN_y} k_{li}^z A_{ij}^x \left\{v_z^0[j] + u_N[j]\right\}
\end{aligned} \tag{S49}$$

and

$$\begin{aligned}
&\langle u_z\rangle = \\
&\frac{F_N + \sum_{i=1}^{N_xN_y}\sum_{j=1}^{N_xN_y} k_{ij}^z[i]u_z^0[j] - \sum_{i=1}^{N_xN_y}\sum_{j=1}^{N_xN_y}\sum_{l=1}^{N_xN_y} k_{li}^z A_{ij}^x \left\{v_z^0[j] + u_N[j]\right\}}{\sum_{i=1}^{N_xN_y}\sum_{j=1}^{N_xN_y} A_{ij}^x k_{ii}^z}.
\end{aligned} \tag{S50}$$

This completes the solution of the model: indeed inserting Eqs.(S48) and (S50) in Eqs.(S41) and (S42) respectively, we calculate the displacement fields at any point (x, y) of the contact plane.

V. Quasi-static dynamics

In this section we discuss the quasi-static dynamics for the discrete elastic model. The adiabatic parameter is the shear force F_S (F_S^{lat}, F_S^{top} or both), and the dynamical protocol is enforced as schematically shown in the flowchart in Fig.S11.

First, we set the value of the normal force F_N and choose a set of $u_z^0[i]$ ($u_z^0[i]$ are sampled from a Gaussian distribution, see appendix VI). Second, we find the value of $\langle u_z\rangle$ according to Eq.(S50) and we plug it into Eq.(18): this gives the set of equilibrium displacements $\vec{u}_z$, satisfying the second condition in Eq.(S43). It is possible that in some point $u_z[i] > u_z^0[i]$, which physically means that the slider $i-$th bottom plane element is not in contact with the underlying rough surface, i.e. the spring is *detached* and the corresponding values of the interfacial stiffnesses $k_x[i]$ and $k_z[i]$ are set to 0, as well as the matrix elements k_{ii}^z and k_{ii}^x. Third, a small shear force F_S is applied: we then calculate the equilibrium configuration along x Eq.(17) using Eq.(S48).

Let us introduce the short notations for the internal stresses at the interface

$$\begin{aligned}
|\tau_{surf}(x,y)| &= |\sigma_{xz}(x,y,0)| = \left|-\varphi_{surf}^x(x,y)\right| = k_x(x,y)\,|u_x(x,y,0)| \\
|\sigma_{surf}(x,y)| &= |\sigma_{zz}(x,y,0)| = \left|-\varphi_{surf}^z(x,y)\right| = k_z(x,y)\,|u_z^0(x,y) - u_z(x,y,0)|\,.
\end{aligned} \tag{S51}$$

After discretizing the contact plane in a mesh, i.e. $\tau_{surf}(x,y) \to \tau_{surf}[i]$ and $\sigma_{surf}(x,y) \to \sigma_{surf}[i]$ (see Eqs.(S93) and (S95)), the local friction law (19) requires that any site i undergo the macroscopic laws of friction. In particular whenever the discrete condition (S96)

$$|\tau_{surf}[i]| \geq \mu\, |\sigma_{surf}[i]| \tag{S52}$$

is satisfied, the corresponding spring is considered *broken*: $k_x[i] = k_z[i] = 0$, $k^z_{ii} = k^x_{ii} = 0$. Althought the mathematical conditions for a spring to be broken or detached are the same, they are dynamically different as the following analysis is going to show.

Given the equilibrium configurations $\vec{u}_z$ and $\vec{u}_x$ for the initial values of F_N and F_S, we start increasing the shear force on the slider. We want to determine the smallest next value of F_S at which one of the *attached* sites for which $k_x[i] \neq 0$ ($k_z[i] \neq 0$) fulfills the condition in Eq.(S52). For such a purpose we first multiply the shear force by a factor $\lambda[i] > 1$:

$$\begin{aligned} F_S^{top} &\to \lambda[i] F_S^{top} \\ u_S^{top} &\to \lambda[i] u_S^{top} \\ F_S^{lat} &\to \lambda[i] F_S^{top} \\ u_S^{lat} &\to \lambda[i] u_S^{lat}. \end{aligned} \tag{S53}$$

Then we insert the previous relations in Eq.(S48), achieving

$$\langle u_x \rangle \to \lambda[i] \langle u_x \rangle. \tag{S54}$$

Plugging Eqs.(S53) and (S54) in (17),

$$u_x[i] \to \lambda[i] u_x[i]. \tag{S55}$$

Now, the requirement Eq.(S52) translates to

$$\lambda[i] = \mu \frac{k_z[i]\,(u_z^0[i] - u_z[i])}{k_x[i]\,|u_x[i]|}, \tag{S56}$$

yielding the set of $\lambda[i]$ at which any i-th spring will be breaking. Hence we take the smallest $\lambda[i] > 1$ and we increase F_S according to Eq.(S53): the corresponding site will be considered broken (putting $k_x[i] = k_z[i] = 0$) and erased from the list of the *available springs*, i.e. those attached, for which $k_z[i] \neq 0$ and $k_x[i] \neq 0$, and the detached ones ($k_x[i] = k_z[i] = 0$, $u_z[i] > u_z^0[i]$ which not fulfilling Eq.(S52)). With this new list of available springs, we

proceed again with the equilibration along z Eqs.(S50)-(18). We notice that some of the detached sites for which previously $u_z[i] > u_z^0[i]$, could now attach ($u_z[i] < u_z^0[i]$): for these we put $k_x[i] = c_x e^{-\frac{u_z[i]}{u_z^0[i]}}$ and $k_z[i] = \frac{c_z}{u_z^0[i]}$ according to (S30); to the contrary, some of the formerly "attached" site could now detach: for these $k_x[i] = k_z[i] = 0$. In either case, a new equilibrium along z is needed. When the values of $\vec{k_x}$ and $\vec{k_z}$ in the list of available springs has not changed, we proceed further to the equilibration along x Eqs.(S48)-(17). After the equilibrium has been reached, it is still possible that one of the attached sites could now fulfill the friction law Eq.(S52) and get broken; if more than one site satisfy the rupture condition, we erase that with the largest elastic energy $\frac{1}{2}k_x[i]u_x[i]^2$. Hence, since the list of available springs has newly changed, we go back to the equilibration along z. It is important to notice that we do not increase further F_S untill the list of available springs has not been modified by any of the possible events: attachment, detachment or rupture. When none of these occur, F_S is further increased according to Eq.(S56) and the quasi-static protocol is repeated until all the springs are broken.

VI. Model calibration

In this section we describe the parameters that enter our model. They can be divided into three sets: *material parameters*, *sample parameters* and *adjustable parameters*.

- *Material parameters.* To this set belong the Young's modulus E, the Poisson's ratio ν and the friction coefficient μ.

  In the case of PMMA $E = 3.833 \times 10^9 N$, $\nu = 0.38$, $\mu = 0.5$.

- *Sample parameters.* To this set belong the macroscopic dimension of the sliders L_x, L_y and L_z, the loading force F_N and the parameters concerning the lateral shear F_S^{lat}: h and Δh.

- *Adjustable parameters.* To this set belong the parameters that characterize the frictional properties of the slider-bottom plane interactions in our model. They are not directly inferred from the experimental setup, since they depend on to our main assumption that surface forces are purely elastic; however we can adjust them in order to reproduce the experimental features of Ref.[6]. They are the mesh sizes Δx and Δy

which set the degree of accuracy in the description of the contact plane slider-rough surface; c_x and c_z which will set the stregth of the linear interactions between the slider and the underlying surface along the x and z directions respectively; the "noise" along the z direction $\vec{u}_z^0$, i.e. the springs rest lengths: this is independent of the slider properties but depends only on the surface roughness.

The following discussion will only concern the set of *adjustable parameters*. The value of Δx and Δy are crucial for the computing time. As a matter of fact, from Eq.(S26), the smaller the mesh sizes, the higher will be the number of elements N_x and N_y, increasing considerably the number of the vector and matrix components appearing in our analysis.
The values of $\vec{u}_z^0$ will be set as follows. Firstly we notice that it corresponds to the average "height" that the underlying rough surface attains within the area $\Delta x \times \Delta y$ centered around the point (x, y) (see Fig.S1). In general (see by instance Ref. [7]) a rough surface is self-affine on distances $\ll \xi$, i.e. given two points (x, y) and (x', y') they are correlated whenever $\sqrt{(x - x')^2 + (y - y')^2} \ll \xi$, where ξ is the surface correlation length. As a consequence of this, the noise $u_z^0(x, y)$ is a correlated Gaussian noise on scales $\ll \xi$, but is an uncorrelated Gaussian noise for distances $\gg \xi$. Throughout our analysis we take $\frac{\Delta x}{\xi} = \frac{\Delta y}{\xi} \gg 1$, since we assume $\xi \simeq 10\mu$ for PMMA and having chosen $\Delta x = \Delta y = 1$mm. We can thus consider the components $u_z^0[i]$ as uncorrelated and Gaussian distributed according to

$$P(u_z^0[i]) = \frac{e^{-\frac{(u_z^0[i] - \langle u_z^0 \rangle)^2}{2\sigma_0^2}}}{\sqrt{2\pi\sigma_0^2}} \theta\left(u_z^0[i]\right). \tag{S57}$$

The average height $\langle u_z^0 \rangle$ is the root mean square surface roughness $\sqrt{w^2}$, while the distribution variance is $\sigma_0^2 = w^2$, being $\sqrt{w^2} \simeq 1\mu$ [8]
Finally we consider both constants c_x and c_z. To set the the constant c_z we refer to the experiment reported in Ref.[6]where it was shown that the real contact area increases linearly with the applied load

$$A_R = \int_0^{L_x} dx \int_0^{L_y} dy\, \rho(x, y) = \alpha F_N \tag{S58}$$

where the constant α can be determined from the experimental curves (see Fig.1(b)), and $\rho(x, y)$ represents the real contact area density. According to the assumption that each surface element behaves as a macroscopic object, we can assume that Eq.(S58) is valid locally as

$$A_R(x,y) \propto \Delta x \Delta y e^{-\frac{u_z(x,y,0)}{u_z^0(x,y)}} \theta\left(u_z^0(x,y) - u_z(x,y,0)\right) \tag{S59}$$

thanks to Eq.(S16). Now, we have to enforce a limit condition for the local real area of contact $A_R(x,y)$: first we want that the area is exactly 0 when $u_z^0(x,y) = u_z(x,y,0)$, hence

$$A_R(x,y) = \gamma \Delta x \Delta y \left[e^{-\frac{u_z(x,y,0)}{u_z^0(x,y)}} - e^{-1}\right] \theta\left(u_z^0(x,y) - u_z(x,y,0)\right) \tag{S60}$$

with $\gamma > 0$. Then we want that for $u_z(x,y,0) = 0$ $A_R(x,y) = \Delta x \Delta y$, which gives $\gamma = \frac{e}{e-1}$

$$A_R(x,y) = \frac{\Delta x \Delta y}{[e-1]} \left[e^{-\frac{u_z(x,y,0) - u_z^0(x,y)}{u_z^0(x,y)}} - 1\right] \theta\left(u_z^0(x,y) - u_z(x,y,0)\right). \tag{S61}$$

In Movie S1 we show the adiabatic evolution of the real contact area for three different loading conditions. Relation (S58) can be cast in the following form after discretizing the contact plane

$$\sum_{i=1}^{N_x N_y} \frac{\Delta x \Delta y}{[e-1]} \left[e^{-\frac{u_z[i] - u_z^0[i]}{u_z^0[i]}} - 1\right] \theta(u_z^0[i] - u_z[i]) = \alpha F_N. \tag{S62}$$

By tuning the value of c_z, we change the equilibrium values $\vec{u}_z$ given by Eqs.(18)-(S50), in order to fulfill Eq.(S62) (see Fig.2(a)). The best value that we found is $c_z = 1.65 \times 10^8 \frac{N}{m^2}$. In Fig.S2 we show the variation of the real area of contact during the shearing process. Fig.S3 shows the same quantity for samples with different nominal area $A_0 = L_x \times L_y$, undergoing the same loading F_N.

Finally, we set c_x by varying the stiffness of the springs along the x direction for the optimal reproduction of the precursors quasi static dynamics for a given sample, as shown in Fig.2(b). The best fit was achieved for $c_x = 1.65 \times 10^{12} \frac{N}{m^3}$.

VII. Displacement controlled shear

The experiments reported in Ref. [6] are performed by applying a lateral shear force through a spring with elastic constant $K_S = 4 \times 10^6$N/m. Hence the external force appearing in Eq.(4) is replaced by Eq. (22). We can thus write the solutions for the displacements at the slider bottom plane as

$$u_x(x,y,0) = \langle u_x\rangle + \frac{(1+\nu)}{E}\left\{\int_0^{L_x} d\xi \int_0^{L_y} d\eta G(x,\xi;y,\eta;0,0)\varphi^x_{surf}(\xi,\eta,0) + \right. \\ \left. + \frac{K_S(U_s-\langle u_x\rangle)}{L_y 2\Delta h}\int_0^{L_y} d\eta \int_{h-\Delta h}^{h+\Delta h} d\zeta G(x,0;y,\eta;0,\zeta)\right\}, \tag{S63}$$

and adopt the shorthand notation

$$K_S(x;h,\Delta h) = \frac{K_S}{L_y 2\Delta h}\int_0^{L_y} d\eta \int_{h-\Delta h}^{h+\Delta h} d\zeta G(x,0;y,\eta;0,\zeta), \tag{S64}$$

where the value of the integral is furnished in Eq.(S22). Notice that in this case we consider only the lateral contribution to the external shearing force, neglecting, for the moment, the force exerted on top of the slider. Once we apply the discretization scheme at the frictional interface, the displacements along $\hat{x}$ become

$$u_x[i] = -\frac{(1+\nu)}{E}\sum_{j=1}^{N_x N_y} \Delta x \Delta y G_{ij} k_x[j] u_x[j] + K_S[i]\left(U_s - \langle u_x\rangle\right) + \langle u_x\rangle, \tag{S65}$$

which in vectorial form read

$$\vec{u}_x = -\frac{(1+\nu)}{E}\hat{G}\hat{k}^x \vec{u}_x + \vec{K}_S\left(U_s - \langle u_x\rangle\right) + \vec{\langle u_x\rangle} \tag{S66}$$

where we implicitely made use of Eq.(S37). Inverting Eq.(S66) we finally achieve

$$\vec{u}_x = \left[\hat{I} + \frac{(1+\nu)}{E}\hat{G}\hat{k}^x\right]^{-1}\left\{\vec{K}_S\left(U_s - \langle u_x\rangle\right) + \vec{\langle u_x\rangle}\right\}, \tag{S67}$$

where we recall that $\vec{\langle u_x\rangle}$ is a vector with all components constant.

The force balance in Eq.S43 now takes te form

$$\sum_{i=1}^{N_x N_y} \Delta x \Delta y k_x[i] u_x[i] = K_S\left(U_s - \langle u_x\rangle\right). \tag{S68}$$

By inserting Eq.(S67) into Eq.(S68) and making use of the defintion Eq.(S44) we obtain, after straightforward passages,

$$\langle u_x\rangle = U_S \frac{\left\{K_S - \sum_{l=1}^{N_x N_y}\sum_{i=1}^{N_x N_y}\sum_{j=1}^{N_x N_y} k^x_{li} A^x_{ij} K_S[j]\right\}}{\left\{K_S - \sum_{l=1}^{N_x N_y}\sum_{i=1}^{N_x N_y}\sum_{j=1}^{N_x N_y} k^x_{li} A^x_{ij} K_S[j] + \sum_{i=1}^{N_x N_y}\sum_{j=1}^{N_x N_y} A^x_{ij} k^x_{ii}\right\}}. \tag{S69}$$

This complete the solution, indeed after Eq.(S69) has been plugged into Eq.(S67) the set of the displacements at the frictional interfaces can be obtained.

VIII. Calculation of Coulomb stress

The evaluation of the normal and tangential stresses on a generic point of the sample (x, y, z) is given by the following expressions

$$\begin{aligned} |\tau(x,y,z)| &= |\sigma_{xz}(x,y,z)| = \tfrac{E}{(1+\nu)}\frac{\partial u_x}{\partial z} \\ |\sigma(x,y,z)| &= |\sigma_{zz}(x,y,z)| = \tfrac{E}{(1+\nu)}\frac{\partial u_z}{\partial z}. \end{aligned} \tag{S70}$$

We start our analysis from the shear internal stress $\tau(x, y, z)$. Inserting Eq.(4) in Eq.(S70) immediately reveals that we need to calculate the derivative of the Green function at (x, y, z):

$$\begin{aligned} \tau(x,y,z) = \Big\{ & \textstyle\int_0^{L_x} d\xi \int_0^{L_y} d\eta \frac{\partial G(x,\xi;y,\eta;z,0)}{\partial z} \varphi^x_{surf}(\xi,\eta,0) - \\ & -\tfrac{F_s^{top}}{L_x L_y} \textstyle\int_0^{L_x} d\xi \int_0^{L_y} d\eta \frac{\partial G(x,\xi;y,\eta;z,L_z)}{\partial z} + \\ & + \textstyle\int_0^{L_y} d\eta \int_{h-\Delta h}^{h+\Delta h} d\zeta \frac{\partial G(x,0;y,\eta;z,\zeta)}{\partial z} \frac{F_s^{lat}}{L_y 2\Delta h} \Big\}. \end{aligned} \tag{S71}$$

The first derivative appearing in the former expression reads

$$\begin{aligned} \tfrac{\partial G(x,\xi;y,\eta;z,0)}{\partial z} = & -\tfrac{8}{L_x L_y L_z^2} \Bigg\{ \sum_{n=1} \frac{n\sin\left(\frac{n\pi z}{L_z}\right)}{4\left(\frac{n\pi}{L_z}\right)^2} + \\ & + \sum_{m=1}\sum_{n=1} \frac{n\sin\left(\frac{n\pi z}{L_z}\right)\cos\left(\frac{m\pi y}{L_y}\right)\cos\left(\frac{m\pi\eta}{L_y}\right)}{2\left[\left(\frac{m\pi}{L_y}\right)^2 + \left(\frac{n\pi}{L_z}\right)^2\right]} + \\ & + \sum_{n=1}\sum_{p=1} \frac{n\sin\left(\frac{n\pi z}{L_z}\right)\cos\left(\frac{p\pi x}{L_x}\right)\cos\left(\frac{p\pi\xi}{L_x}\right)}{2\left[\left(\frac{n\pi}{L_z}\right)^2 + \left(\frac{p\pi}{L_x}\right)^2\right]} + \\ & + \sum_{m=1}\sum_{n=1}\sum_{p=1} \frac{n\sin\left(\frac{n\pi z}{L_z}\right)\cos\left(\frac{m\pi y}{L_y}\right)\cos\left(\frac{m\pi\eta}{L_y}\right)\cos\left(\frac{p\pi x}{L_x}\right)\cos\left(\frac{p\pi\xi}{L_x}\right)}{\left(\frac{n\pi}{L_z}\right)^2 + \left(\frac{n\pi}{L_z}\right)^2 + \left(\frac{m\pi}{L_y}\right)^2 + \left(\frac{p\pi}{L_x}\right)^2} \Bigg\}. \end{aligned} \tag{S72}$$

The first sum in Eq.(S72) can be evaluated according to Ref. [2] as:

$$\frac{1}{4}\sum_{n=1} \frac{n\sin\left(\frac{n\pi z}{L_z}\right)}{\left(\frac{n\pi}{L_z}\right)^2} = L_z \frac{(L_z - z)}{8\pi}, \tag{S73}$$

and the second can be rewritten in the form

$$\begin{aligned} & \tfrac{1}{2}\sum_{m=1}\sum_{n=1} \frac{n\sin\left(\frac{n\pi z}{L_z}\right)\cos\left(\frac{m\pi y}{L_y}\right)\cos\left(\frac{m\pi\eta}{L_y}\right)}{\left(\frac{m\pi}{L_y}\right)^2 + \left(\frac{n\pi}{L_z}\right)^2} = \\ & \tfrac{L_z}{8\pi} \left\{ \mathscr{G}(|y-\eta|, z; L_y, L_z) + \mathscr{G}(y+\eta, z; L_y, L_z) \right\} \end{aligned} \tag{S74}$$

in terms of the function

$$\mathscr{G}(a,b;L_a,L_b) = L_b \sum_{m=1} \frac{\cos\left(\frac{m\pi a}{L_a}\right)\sinh\left(\frac{m\pi(L_b-b)}{L_a}\right)}{\sinh\left(\frac{m\pi L_b}{L_a}\right)}. \tag{S75}$$

The third term can be treated in the same way. The fourth element in the bracket of Eq.(S72) is easily evaluated:

$$\begin{aligned}
&\sum_{m=1}\sum_{n=1}\sum_{p=1} \frac{n\sin\left(\frac{n\pi z}{L_z}\right)\cos\left(\frac{m\pi y}{L_y}\right)\cos\left(\frac{m\pi\eta}{L_y}\right)\cos\left(\frac{p\pi x}{L_x}\right)\cos\left(\frac{p\pi\xi}{L_x}\right)}{\left(\frac{n\pi}{L_z}\right)^2+\left(\frac{n\pi}{L_z}\right)^2+\left(\frac{m\pi}{L_y}\right)^2+\left(\frac{p\pi}{L_x}\right)^2} = \\
&\tfrac{L_z}{8\pi}\left\{\mathscr{Q}(|x-\xi|,|y-\eta|,z;L_x,L_y,L_z)+\mathscr{Q}(|x-\xi|,y+\eta,z;L_x,L_y,L_z)+\right. \\
&+\mathscr{Q}(x+\xi,|y-\eta|,z;L_x,L_y,L_z)+\mathscr{Q}(x+\xi,y+\eta,z;L_x,L_y,L_z)+ \\
&\left.+\mathscr{Q}(x+\xi,y+\eta,z;L_x,L_y,L_z)\right\}
\end{aligned} \tag{S76}$$

where

$$\begin{aligned}
&\mathscr{Q}(a,b,c;L_a,L_b,L_c) = \\
&L_z \sum_{m=1}\sum_{p=1} \frac{\cos\left(\frac{p\pi a}{L_a}\right)\cos\left(\frac{m\pi b}{L_b}\right)\sinh\left(\frac{\pi(L_c-c)}{L_aL_b}\sqrt{(pL_b)^2+(mL_a)^2}\right)}{\sinh\left(\frac{\pi L_c}{L_aL_b}\sqrt{(pL_b)^2+(mL_a)^2}\right)}.
\end{aligned} \tag{S77}$$

Hence from Eqs.(S73), (S74), (S76), Eq.(S72) transforms to

$$\begin{aligned}
&\tfrac{\partial G(x,\xi;y,\eta;z,0)}{\partial z} = -\tfrac{1}{L_xL_yL_z}\left\{L_z - z + \mathscr{G}(|y-\eta|,z;L_y,L_z)+\right. \\
&+\mathscr{G}(y+\eta,z;L_y,L_z)+\mathscr{G}(|x-\xi|,z;L_x,L_z)+\mathscr{G}(x+\xi,z;L_x,L_z)+ \\
&+\mathscr{Q}(|x-\xi|,|y-\eta|,z;L_x,L_y,L_z)+\mathscr{Q}(|x-\xi|,y+\eta,z;L_x,L_y,L_z)+ \\
&+\mathscr{Q}(x+\xi,|y-\eta|,z;L_x,L_y,L_z)+\mathscr{Q}(x+\xi,y+\eta,z;L_x,L_y,L_z)+ \\
&\left.+\mathscr{Q}(x+\xi,y+\eta,z;L_x,L_y,L_z)\right\}.
\end{aligned} \tag{S78}$$

The second term in Eq.(S71) is straightforwardly evaluated (see [2])

$$\int_0^{L_x} d\xi \int_0^{L_y} d\eta \frac{\partial G(x,\xi;y,\eta;z,L_z)}{\partial z} = \frac{z}{L_z}. \tag{S79}$$

Finally, the third term is

$$\begin{aligned}
&\int_0^{L_y} d\eta \int_{h-\Delta h}^{h+\Delta h} d\zeta \tfrac{\partial G(x,0;y,\eta;z,\zeta)}{\partial z}\Big|_{x,y,z} = \\
&-\tfrac{1}{2L_x}\left\{\tfrac{(z-h-\Delta h)^2-(z+h+\Delta h)^2+(z-h+\Delta h)^2+(z+h-\Delta h)^2}{2L_z} - \right. \\
&\left. -|z-h-\Delta h|+|z+h+\Delta h|-|z-h+\Delta h|+|z-h-\Delta h|\right\} - \\
&-\tfrac{2}{\pi}\sum_{n=1}\tfrac{\cosh\left(\frac{n\pi}{L_z}(L_x-x)\right)}{n\sinh\left(\frac{n\pi L_x}{L_z}\right)}\left\{\cos\left(\tfrac{n\pi}{L_z}|z-h-\Delta h|\right)-\cos\left(\tfrac{n\pi}{L_z}(z+h+\Delta h)\right)+\right. \\
&\left.+\cos\left(\tfrac{n\pi}{L_z}|z-h+\Delta h|\right)-\cos\left(\tfrac{n\pi}{L_z}|z+h-\Delta h|\right)\right\}.
\end{aligned} \tag{S80}$$

Inserting Eqs.(S78), (S79) and (S80) in Eq.(S71) we obtain the expression for the shear stress at any point of the sample.

The normal stress can be written as

$$\begin{aligned}\sigma(x,y,z) = \Big\{ &\textstyle\int_0^{L_x} d\xi \int_0^{L_y} d\eta \frac{\partial G(x,\xi;y,\eta;z,0)}{\partial z} \varphi^z_{surf}(\xi,\eta,0) - \\ &\textstyle - \frac{F_N}{L_x L_y} \int_0^{L_x} d\xi \int_0^{L_y} d\eta \frac{\partial G(x,\xi;y,\eta;z,L_z)}{\partial z} \Big\}\end{aligned} \tag{S81}$$

and can be handled in the same way as the shear stress, plugging Eqs.(S78) and (S79) into Eq.(S81).

In Fig.S10 we show the quasi-static evolution of the normal and shear stress at the reference plane $z_P = 2$mm. We notice that in the limit $z_P \to 0$, Eq.(S71) and Eq.(S81) transform to S51. Finally we can define the Coulomb stress at any point in the sample

$$\tau_C(x,y,z) = |\tau(x,y,z)| - \mu\, |\sigma(x,y,z)| \,. \tag{S82}$$

When we perform the mesh discretization at the contact plane, we have to map the variables on the plane according to Eq.(S29), i.e. $(x,y) \to i$. Hence the derivative of the Green's function Eq.(S78) transforms to the following matrix

$$\frac{\partial G(x,\xi;y,\eta;z,0)}{\partial z} \to \frac{\partial \hat{G}_{ij}}{\partial z}. \tag{S83}$$

The second term in the shear stress (Eq.(S71)) is constant as it results from Eq.(S79), then we can write

$$-\frac{F_s^{top}}{L_x L_y} \int_0^{L_x} d\xi \int_0^{L_y} d\eta \frac{\partial G(x,\xi;y,\eta;z,L_z)}{\partial z} \to \tau_z^{top}[i_P]. \tag{S84}$$

The same can be done for the third term, although from Eq.(S80) it is apparent its dependence on x:

$$\frac{F_s^{lat}}{L_y 2\Delta h} \int_0^{L_y} d\eta \int_{h-\Delta h}^{h+\Delta h} d\zeta \frac{\partial G(x,0;y,\eta;z,\zeta)}{\partial z} \to \tau_z^{lat}[i]. \tag{S85}$$

The discrete form of the shear stress can be cast as

$$\tau_z[i] = -\Delta x \Delta y \sum_{j=1}^{N_x N_y} \frac{\partial \hat{G}_{ij}}{\partial z} \left(k_x[j] u_x[j] + \tau_z^{top}[i] + \tau_z^{lat}[i] \right) \tag{S86}$$

and in the vectorial form

$$\vec{\tau}_z = -\frac{\partial \hat{G}}{\partial z}\left(\hat{k}^x \vec{u}_x + \vec{\tau}_z^{top} + \vec{\tau}_z^{lat}\right) \tag{S87}$$

thanks to Eq.(S37). Applying the same analysis to the normal stress Eq.(S81), we have for the second term the same as Eq.(S84), i.e.

$$-\frac{F_N}{L_x L_y}\int_0^{L_x} d\xi \int_0^{L_y} d\eta \frac{\partial G(x,\xi;y,\eta;z,L_z)}{\partial z} \to \sigma_z^N[i], \tag{S88}$$

and finally

$$\sigma_z[i] = \Delta x \Delta y \sum_{j=1}^{N_x N_y} \frac{\partial \hat{G}_{ij}}{\partial z}\left(k_z[j]\left(u_z^0[j] - u_z[j]\right) + \sigma_z^N[i]\right) \tag{S89}$$

which in the vectorial form transforms to

$$\vec{\sigma}_z = \frac{\partial \hat{G}}{\partial z}\left(\hat{k}^z\left(\vec{u}_z^0 - \vec{u}_z\right) + \vec{\sigma}_z^N\right) \tag{S90}$$

thanks to (S38). Hence the discrete expression of the Coulomb stress is given by

$$\vec{\tau}_z^C = |\vec{\tau}_z| - \mu\, |\vec{\sigma}_z| \tag{S91}$$

and its quasi-static evolution is shown in Movie S2.

IX. Geometrical dependence of front precursors

We report here some general consideration on the role of geometry in the shape of precursors. Plugging the continuous expression of Eq.(17) into the first of Eqs.(S51), we obtain that the shear stress at the interface can be written as

$$\tau_{surf}(x,y) = k_x(x,y)\int_0^{L_x} d\xi \int_0^{L_y} d\eta\, A^x(x,y;\xi,\eta)\left[u_S^{top}(\xi,\eta) + u_S^{lat}(\xi,\eta) + \langle u_x\rangle\right], \tag{S92}$$

or, in its discrete form, as

$$\tau_{surf}[i] = k_x[i]\sum_{j=1}^{N_x N_y} A_{ij}^x\left[u_S^{top}[j] + u_S^{lat}[j] + \langle u_x\rangle\right]. \tag{S93}$$

$A^x(x,y;\xi,\eta)$ is the continuous version of the matrix A_{ij}^x introduced in Eq.(17), connected to the inverse of the Green's operator and to $k_x(x,y)$ (see Eq.(S44)). According to Eq.(S92),

$\tau_{surf}(x,y)$ can be decomposed into three contributions stemming from (i) forces applied to the top surface F_S^{top}, (ii) forces applied on the trailing edge F_S^{lat} and (iii) the average volume shift (see respectively Eqs.(S21), (S24) and Eq.(S48)). Among these, only the second term is responsible for the stress gradient at the interface. Indeed, when loading is exerted on the top plate, $u_S^{lat} = 0$ and u_S^{top} is uniform across the entire slider bottom plane as it turns out from Eqs.(S20) and (S21). Moreover $\langle u_x \rangle$ is constant for any site, being the avergae displacement.

The normal stress at the interface, on the other hand, takes the following continuous and discrete expression respectively

$$\sigma_{surf}(x,y) = k_z(x,y) \left\{ \int_0^{L_x} d\xi \int_0^{L_y} d\eta \, A^z(x,y;\xi,\eta) \left[v_0(\xi,\eta) + u_N(\xi,\eta) + \langle u_z \rangle \right] - u_z^0(x,y) \right\}, \tag{S94}$$

$$\sigma_{surf}[i] = k_z[i] \left\{ \sum_{j=1}^{N_x N_y} A_{ij}^x \left[v_0[j] + u_N[j] + \langle u_z \rangle \right] - u_z^0[j] \right\}. \tag{S95}$$

$A^z(x,y;\xi,\eta)$ and $v_0(x,y)$ represent the continuous expressions of Eqs.(S45) and (S46), $u_N(x,y)$ is introduced in Eq.(S25) and the average shift expression $\langle u_z \rangle$ is defined in its discrete form in Eq.(S50). The three terms in Eq.(S94) do not exhibit any apparent gradient on the slider bottom plane, since they depend on the material bottom surface heterogeneity ($v_0(x,y)$), which in average provides an uniform contribution; on the normal applied load F_N, which is again even and uniform throughout the frictional interface (the edge effects are playing indeed a minor role); and on $\langle u_z \rangle$ which is by definition uniform.

With the discrete expressions for the surface shear and normal stresses at hand, we can write the local slip condition (19) as

$$\tau_{surf}^C[i] = |\tau_{surf}[i]| - \mu \, |\sigma_{surf}[i]| \,. \tag{S96}$$

Based on the former analysis, when shear is applied uniformly from above, i.e. $F_S \equiv F_S^{top}$, our model suggests that both shear (τ_{surf}) and normal (σ_{surf}) component of the Coulomb stress are uniform on the contact surface and therefore no precursors are expected. This is clearly seen in Fig.3(d) and corroborates the finding of one-dimensional friction models [9]. However, scalar model does just furnish an average picture of the internal stress behaviors

when shear is applied uniformly on the top, as it does not account, by instance, for the elastic Poisson expansion, nor for torque, responsibles for the stresses heterogeneities observed at the sample edges [10].

When the shear force is applied on the trailing edge, i.e. $F_S \equiv F_S^{lat}$, only the second term proportional to u_S^{lat} survives in Eqs.(S92), (S93). Therefore the shear stress τ_{surf} displays an evident gradient across the contact interface, leading to detachment of the regions where this gradient is more pronounced and, eventually, to precursors activity. In particular, when loading uniformly from the trailing edge ($\Delta h = L_z/2$), the corresponding contribution given by u_S^{lat} to τ_{surf} is

$$\tau_{ii}(x,y) = \frac{(1+\nu)}{E}\frac{L_x}{L_y L_z} F_s^{lat} k_x(x,y)\left[\frac{x^2}{2L_x^2} - \frac{x}{L_x} + \frac{1}{3}\right], \tag{S97}$$

and the interface shear stress dependence on the sample geometry is entirely encapsulated in the ratio $R = \frac{L_x}{L_y L_z}$ (see Eqs.(S23), (S24)). Since we argue that the precursors envelope curves reflect the symmetries appering in the Coulomb stress $\tau_C(x,y,0)$, the rescaled precursor profile ℓ/L_x should only depend on R, in complete agreement with the numerics reported in Fig. 6(a),(b),(c).

If F_S^{lat} is applied by a rod at height $z = h$, a direct evaluation of the corresponding of u_S^{lat} contribution to the expressions (S92), (S93) yields a non-trivial dependence on L_x, L_y, L_z, h and Δh, as it is immediate to see by referring to Eqs.(S22) and Eqs.(S24). As a result, no universal scaling on the sample dimensions and rod parameters (h, Δh) can be found in this case, as already illustrated in Figs. 5(b), S4, S5, S6, S7, S8, S9.

When the shear is applied from the slider top and trailing edge simultaneously, both terms u_S^{lat} and u_S^{top} in Eq.(S92) are competing: when the first dominates, no precursor nucleation should be seen and the slider detaches as a whole when the frictional force is reached. This is indeed what is shown in Fig.6(d). Yet, conclusions about uniform top shearing are to be taken with a grain of salt as explained before, indeed they are valid only on average and for very large samples, since no edges nonuniformities are encompassed within the scalar elasticity theory.

Finally, the role of the contact plane disorder heterogeneity on the interfacial stresses appears in Eqs.(S92), (S94) through $A^x(x,y;\xi,\eta)$, $A^z(x,y;\xi,\eta)$, $k_x(x,y)$, $k_z(x,y)$ and $v_0(x,y)$. For the relative narrow distribution of the heights $u_0^z(x,y)$ characterizing the rough substrate here considered, the contribution of the interfacial disorder to the precursor nucleation is

irrelevant and uniform in average.

X. The finite element model

The system has been initially simulated by means of a three-dimensional model based on the finite element method (FEM). In order to compare this model with experiments, we chose parameters that correspond to the PMMA samples employed in Refs. [6, 10–13]. In particular, we make use of the sample geometry and dimensions used in those experiments and of the known elastic constants (Young's modulus E and Poisson's ratio ν) of PMMA. In addition, the slider bottom corners are rounded by a radius of 2 mm to avoid both stress singularities at the edges and frustrated Poisson expansion [6, 10, 14].

For the simulations we used the commercial FEM software COMSOL. We approximated the geometry and displacements by use of quadratic shape functions and chose the size and local refinement of the tetrahedral finite element mesh such that further refinement would give no appreciable gain in accuracy. Resulting displacements within elements were interpolated using the polynomials of the shape functions, stresses and strains were interpolated using gradients of the shape functions.

A. Model calibration and stress profiles

Our model encompasses the setting of only two parameters, i.e. the asperity spring stiffnesses k_x and k_z, as the material elastic constants have been chosen to reproduce the PMMA properties. To tune the values of k_x and k_z our benchmark is the experiment of Ref.[10], where the internal normal and shear stresses, $\sigma(x, y, z)$ and $\tau(x, y, z)$ respectively, were calculated at the points $(x, y = L_y/2, z = z_p)$ where z_p is a plane placed 2mm above the bottom surface. The slider dimensions were $L_x = 200mm$, $L_y = 6mm$ and $L_z = 100mm$ while no shearing force was applied on the PMMA block. We have reproduced the experimental setup by means of our FEM model, and adjusted the values of k_x and k_z until the shear and normal stress profiles respectively were showing a satisfactory agreement: the results are displayed in Fig.S13. In particular, it is possible to see how our 3D FEM model not only furnishes an accurate quantitative agreement with the experimental curves (dashed lines), but accounts also for the high nonuniformity exhibited by normal and shear

stress when no shearing is present. Indeed $\sigma(x, L_y/2, z_p)$ clearly displays divergences at the bottom edges due to the interface pinnning, as well as $\tau(x, L_y/2, z_p)$. In this regard, in the experiments reported in [10, 13], shear stress nonuniformity deriving from differential Poisson expansion frustrated at the interface, was reduced by leaving the block edges free to expand. In our model, both normal and shear stress nonuniformities could be controlled thanks to the rounding of the block's bottom corners, providing a remarkable agreement of the stress profiles at $F_S = 0$.

The situation considerably changes when $F_S \neq 0$. Experimentally, normal stress $\sigma(x, L_y/2, z_p)$ shows stronger nonuniformities localized to regions near the block's edges, as the effect of the torque induced by the application of F_S(see Fig.S14(a) and (c), dashed lines). This effect is much more pronounced when shear is applied at the sample's trailing edge ($F_S = F_S^{lat}$), by means of a 4mm-sized rod placed at height $h = 6$mm [10] (Fig.S14(a)). To partially avoid this byproduct, a controlled gradient in F_N was applied by introducing a slight rigid tilt to the PMMA slider. This rigid tilt of the block was notably enhanced when shear was applied on the side. We could not reproduce such a tilt in our FEM model, since this loading controlled gradient leads to a highly non-linear system response, causing severe stability problems during the FEM solution. Furthermore, also the shear stress $\tau(x, L_y/2, z_p)$ is considerably altered when $F_S \neq 0$ (see Fig.S14(b) and (d), dashed lines). As in the case of σ, τ nonuniformities appear more marked while shear is impressed from aside (Fig.S14(b), dashed line) instead of uniformly on the block's top (Fig.S14(d), dashed line).

While trying to reproduce the internal stress profiles for $F_S \neq 0$, our FEM model fails. Indeed none of the simulated stresses, achieved for different values of the shearing force and different modes of shearing, exhibits a satisfactory agreement with the experimental curves, although qualitatively the observed trends are respected (see FigS14). We believe that our model failure is ultimately ascribable to the impossibility of transposing numerically the tilting block expedient employed in the experiment. As a matter of fact, when F_S is applied uniformly on top of the slider, the agreement between experimental and numerical stress profiles appear to be slightly better than when shear is imposed from the edge.

---

[1] S. Marshall, Proc. R. Soc. Lond. A **455**, 1739 (1999).

[2] A. P. Prudnikov, I. A. Brychkov, and O. I. Maricev, *Integrals and Series*, 1st ed., Vol. 1 (Nauka, Moscow, 1981).

[3] M. Benz, K. J. Rosenberg, E. J. Kramer, and J. N. Israelachvili, J Phys Chem B **110**, 11884 (2006).

[4] B. N. J. Persson, Phys. Rev. Lett. **99**, 125502 (2007).

[5] P. Berthoud and T. Baumberger, Proc. R. Soc. Lond. A **454**, 1615 (1998).

[6] S. M. Rubinstein, G. Cohen, and J. Fineberg, Phys. Rev. Lett. **96**, 256103 (2006).

[7] B. Persson, Surface Science Reports **61**, 201 (2006).

[8] S. Rubinstein, M. Shay, G. Cohen, and J. Fineberg, International Journal of Fracture **140**, 201 (2006).

[9] R. Capozza and M. Urbakh, Phys. Rev. B **86**, 085430 (2012).

[10] O. Ben-David, G. Cohen, and J. Fineberg, Science **330**, 211 (2010).

[11] S. M. Rubinstein, G. Cohen, and J. Fineberg, Nature **430**, 1005 (2004).

[12] S. M. Rubinstein, G. Cohen, and J. Fineberg, Phys. Rev. Lett. **98**, 226103 (2007).

[13] O. Ben-David and J. Fineberg, Phys. Rev. Lett. **106**, 254301 (2011).

[14] M. Radiguet, D. S. Kammer, P. Gillet, and J.-F. m. c. Molinari, Phys. Rev. Lett. **111**, 164302 (2013).

[15] J. A. Greenwood and J. B. P. Williamson, Proc. R. Soc. Lond. A **295**, 300 (1966).

Supplementary movies

Movie S1: Evolution patterns of the real contact area during the shear adiabatic protocol. Different loading conditions are applied to the same sample: $F_N = 2.7$kN, $L_x = 201$mm, $L_y = 7$mm, $L_z = 75$mm ($h = 6$mm and $\Delta h = 2$mm when the shearing is applied with a rod). The real contact area is normalized to the first photogram for which $F_S = 0$. Pixels represent the mesh of the contact plane, each pixel having a physical area of $\Delta x \times \Delta y = 1mm^2$. The color code goes from dark blue ($A_R(F_S)/A_R(0) \ll 1$) to white ($A_R(F_S)/A_R(0) \gg 1$). However, due to the large fluctuations of the normalized real contact area during the precursor dynamics, the color mapping is costantely changed to appreciate the varying range of $A_R(F_S)/A_R(0)$.

Movie S2: Coulomb stress and relative real contact area during the shear adiabatic protocol. (a) Quasi-static dynamics of the Coulomb stress defined as $\tau_C(x,z) = \left|\frac{\int_0^{L_y} dy\, \tau(x,y,z)}{L_y}\right| - \mu\left|\frac{\int_0^{L_y} dy\, \sigma(x,y,z)}{L_y}\right|$, with $\tau(x,y,z)$ and $\sigma(x,y,z)$ from Eq.S71 and Eq.S81 respectively. The color code goes from dark blue ($\tau_C \ll 0$) to red ($\tau_C \gg 0$), grey line represents the evolution of the set of points fulfilling $\tau_C = 0$. Sample is subjected to a double shearing $F_S = F_S^{top} + F_S^{lat}$: with a rod ($h = 6$mm, $\Delta h = 2$mm) and uniformly from the sample top layer. Other sample parameters are $F_N = 6.25$kN, $L_x = 200$mm, $L_y = 7$mm, $L_z = 100$mm. Each photogram is averaged over 10 independent realizations. (b) Evolution pattern of the real contact area, normalized to the first photogram, for the same sample shown in panel (a). Here however, no average is shown, but a single realization only. Color code goes from dark blue ($A_R(F_S)/A_R(0) \ll 1$) to white ($A_R(F_S)/A_R(0) \gg 1$). Due to the large fluctuations of the normalized real contact area and τ_C during the precursor dynamics, the color mapping is costantely changed to appreciate the varying range of both observables.

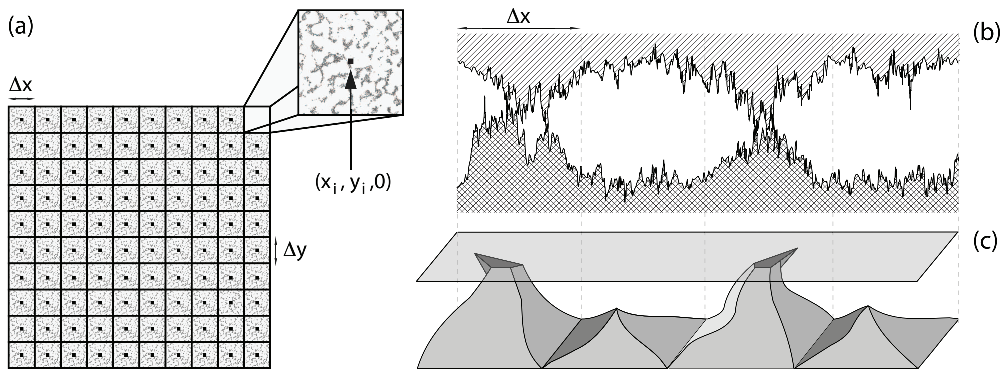

FIG. S1. **Discretization**. Mesh of the slider-rough surface contact plane (a). The central point of each mesh element is representative of the microscopical roughness-induced forces within the interface portion $\Delta x \times \Delta y$ (b). Panel (c): coarse-grained asperity-like interaction of the substrate with the upper slider. The elastic picture emerging in panel (c) is mathematically translated in sthe pring-like interaction as shown in Fig.1 (bottom panel): the slider lais on a carpet of springs $u_z^0[i]$.

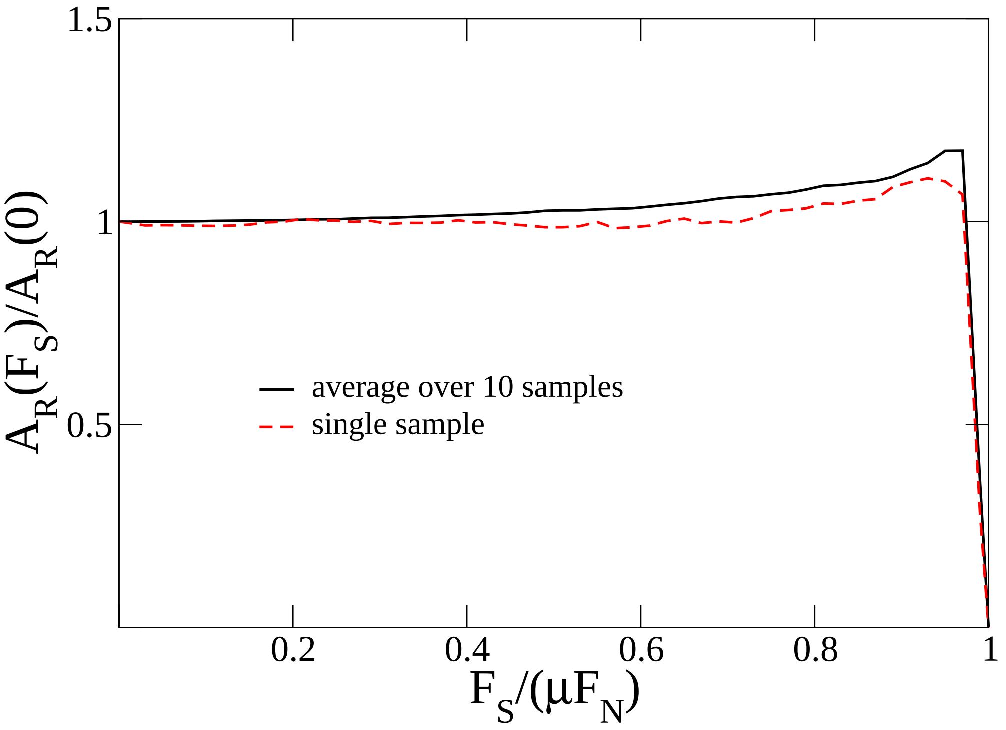

FIG. S2. **Area of real contact during slip**. Variation of the real area of contact (normalized to the initial value) as a function of the shearig F_S. Sample parameters are $L_x = 100$mm, $L_y = 7$mm, $L_z = 75$mm, $F_N = 4$kN. Uniform top shear is applied, i.e. $F_S \equiv F_S^{top}$

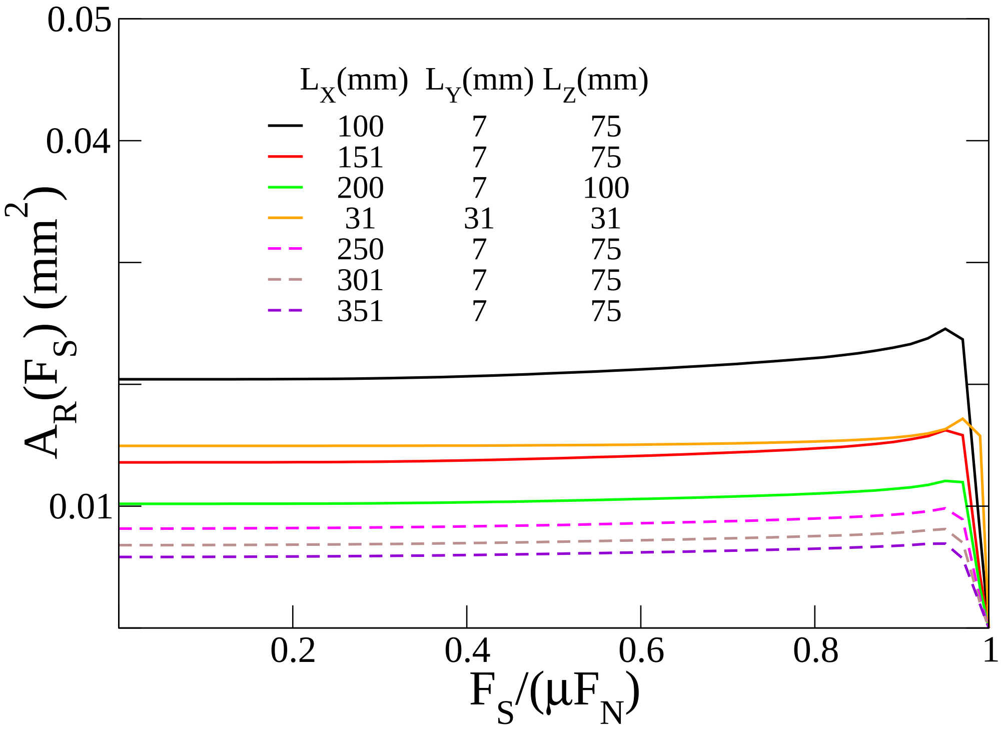

FIG. S3. **First and second Amontons' laws**. Real area of contact A_R during the shearing process for various samples, $F_N = 4$kN. Althoug nominal area $A_0 = L_x \times L_y$ are very different for the shown samples, A_R appears to be only dependent on the load F_N and independent from the nominal area of contact, i.e. $A_R = \alpha F_N$ [15] (this can be also seen in Fig.2(a)). Small variations of A_R are persistent in different samples as shown in the figure, they are due mainly to the mesh discretization. Solid lines correspond to a uniform lateral shearing from the sample trailing edge, dashed lines correspond to a lateral shearing with a rod at height $h = 6$mm ($\Delta h = 2$mm). Plots are averaged over 10 independent realizations. Since the sliding happens *always* at $\frac{F_S}{\mu F_N} = 1$ the first Amontons' law is satisfied. Moreover, since A_R solely depends on F_N, the frictional force F_S at which the sliding takes place is only dependent on A_R and not on A_0, what goes under the name of Amontons' second law.

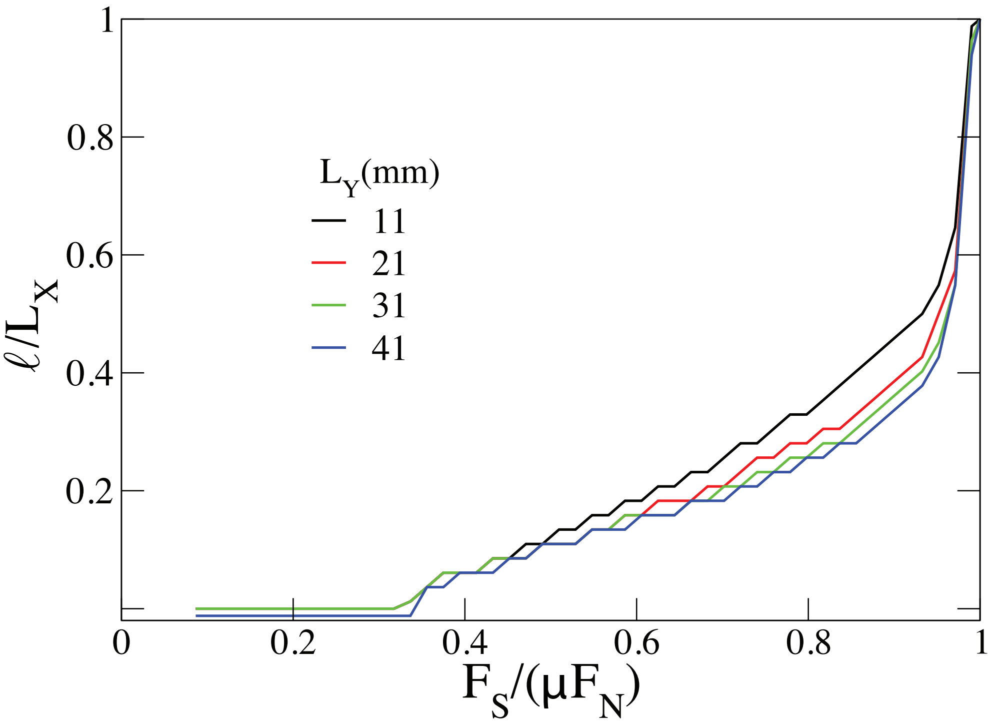

FIG. S4. **The dependence of slip precursors on the sample size** L_y. Precursor quasi-static dynamics for different L_y. The sample is sheared with a rod at the height $h = 6$mm ($\Delta h = 2$mm), other sample parameters are $F_N = 4$kN, $L_x = 41$mm, $L_z = 132$mm. Plots are averaged over 10 independent realizations.

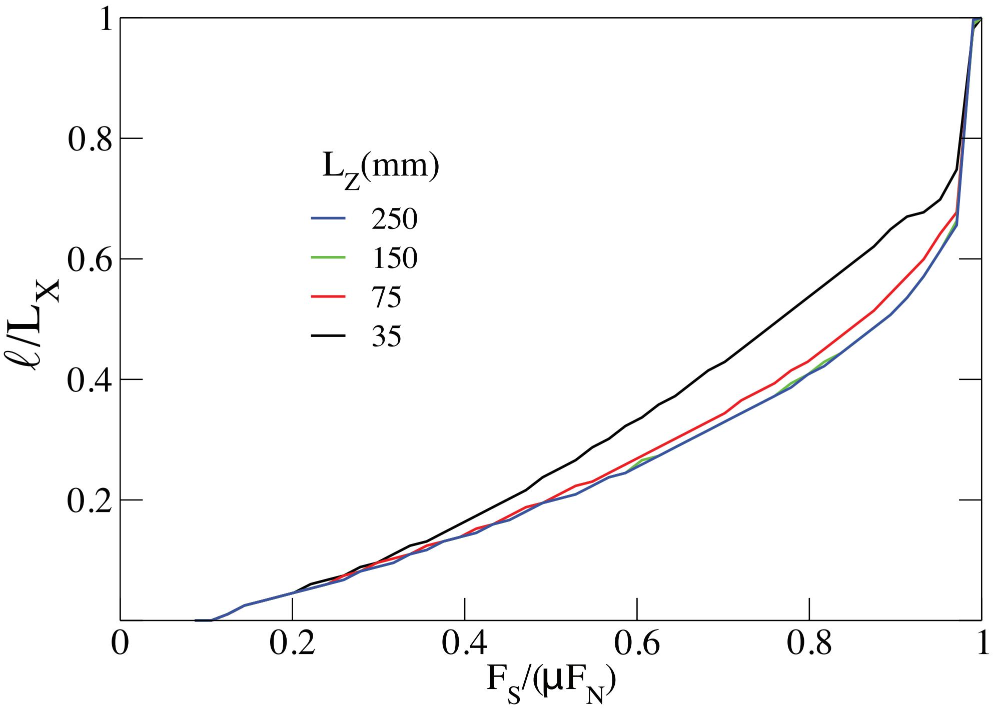

FIG. S5. **The dependence of slip precursors on the sample size** L_z. Precursor quasi-static dynamics for different L_z. The sample is sheared with a rod at the height $h = 6$mm ($\Delta h = 2$mm), other sample parameters are $F_N = 4$kN, $L_x = 141$mm, $L_y = 7$mm. Plots are averaged over 10 independent realizations.

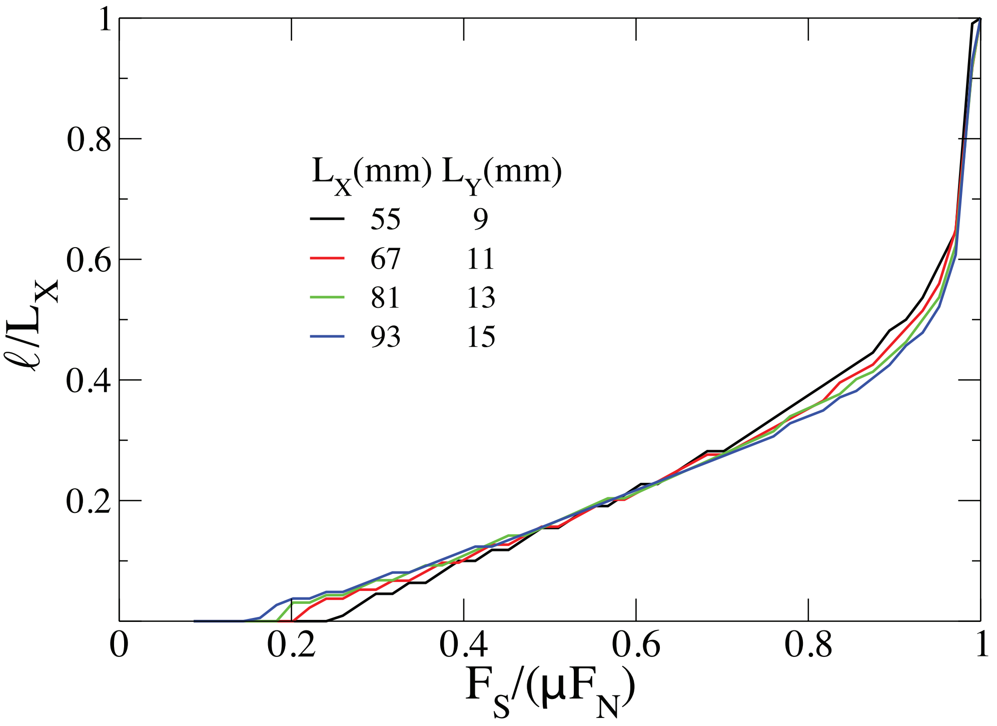

FIG. S6. **The dependence of slip precursors on the sample aspect ratio** L_x/L_y. Precursor quasi-static dynamics for different L_x and L_y but same aspect ratio $\frac{L_x}{L_y} \simeq 6$. The sample is sheared with a rod at the height $h = 6$mm ($\Delta h = 2$mm), other sample parameters are $F_N = 4$kN, $L_z = 100$mm. Plots are averaged over 10 independent realizations.

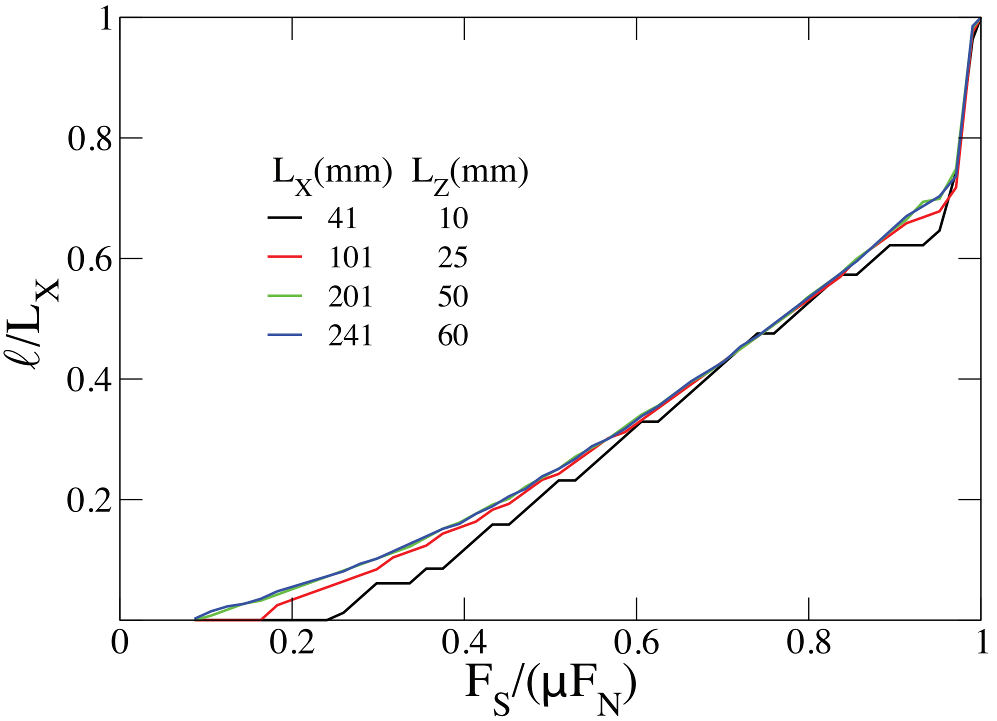

FIG. S7. **The dependence of slip precursors on the sample aspect ratio** L_x/L_z. Precursor quasi-static dynamics for different L_x and L_z but same aspect ratio $\frac{L_x}{L_z} \simeq 4$. The sample is sheared with a rod at the height $h = 6$mm ($\Delta h = 2$mm), other sample parameters are $F_N = 4$kN, $L_y = 7$mm. Plots are averaged over 10 independent realizations.

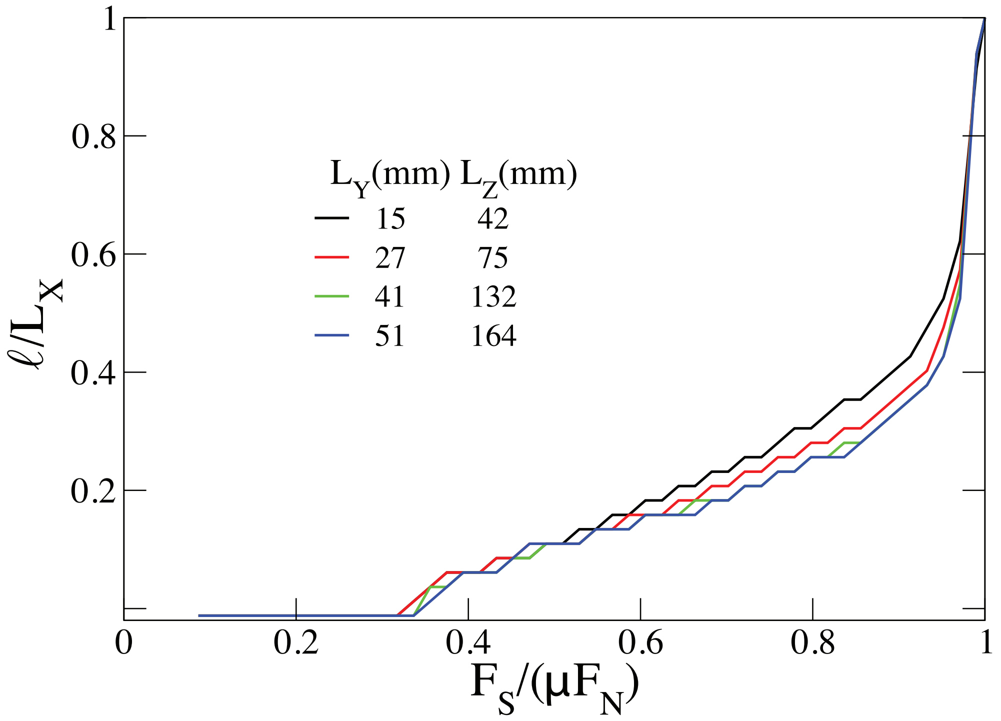

FIG. S8. **The dependence of slip precursors on the sample aspect ratio** L_y/L_z.Precursor quasi-static dynamics for different L_y and L_z but same aspect ratio $\frac{L_y}{L_z} \simeq 0.36$. The sample is sheared with a rod at the height $h = 6$mm ($\Delta h = 2$mm), other sample parameters are $F_N = 4$kN, $L_x = 141$mm. Plots are averaged over 10 independent realizations.

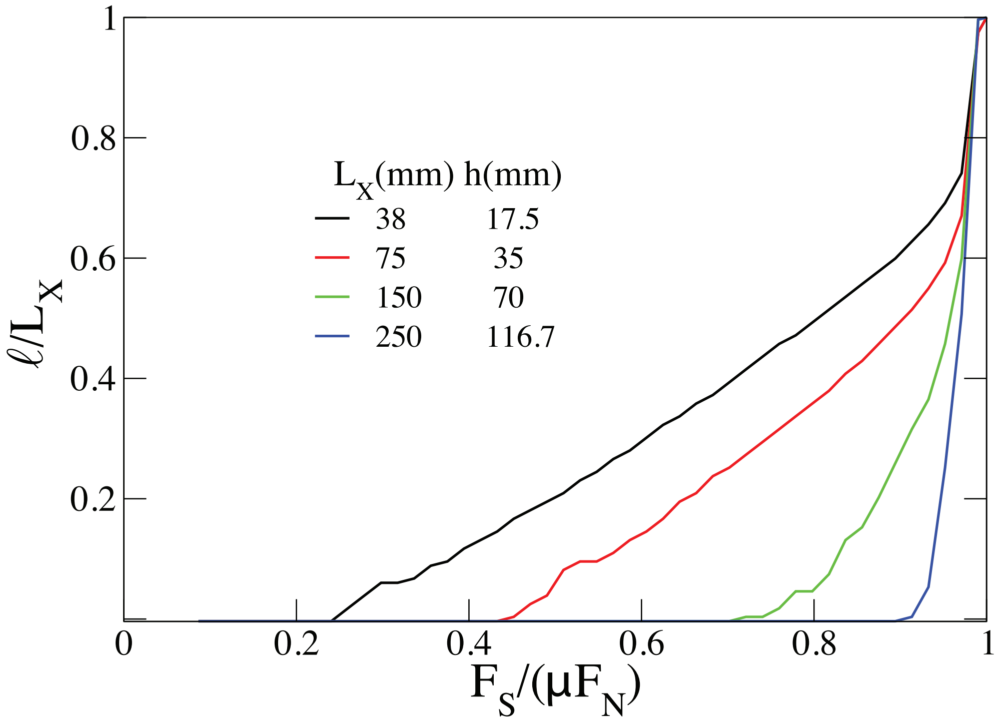

FIG. S9. **The dependence of slip precursors on the ratio** h/L_z. Precursor quasi-static dynamics when the sample undergoes a lateral shearing with a rod at the height $h = 6$mm ($\Delta h =$ 2mm). Plots show different profiles obtained by varying h and L_z but keeping constant the ratio $\frac{h}{L_z} \simeq 0.46$. Other sample parameters are $F_N = 4$kN, $L_x = 141$mm, $L_y = 7$mm. Plots are averaged over 10 independent realizations.

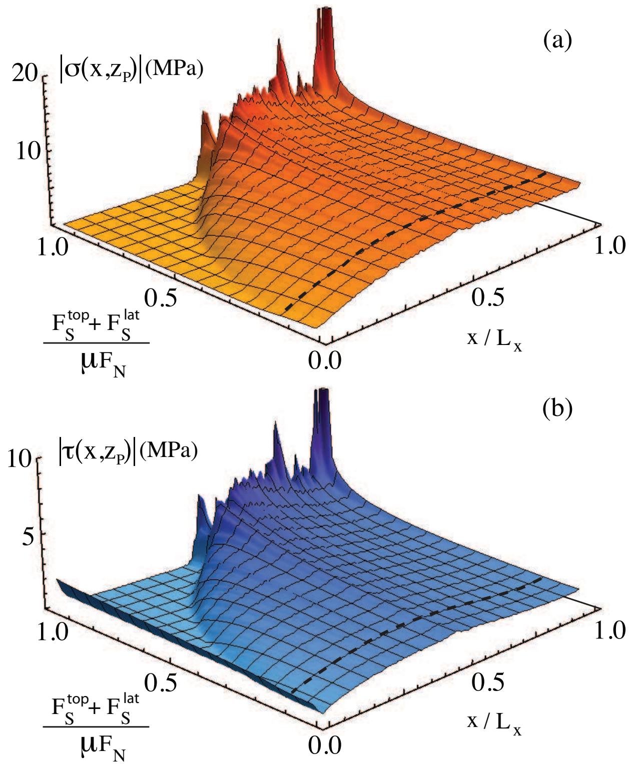

FIG. S10. **Shear and normal stresses at z_P = 2mm. Quasi-static evolution.**Average internal stresses calculated at the reference plane $z_P = 2$mm. (a) Normal stress obtained from Eq.S81 (Eq.S89 in its discrete form) and averaged along the y direction, i.e. $|\sigma(x, z_P)|$ during a shearing protocol which involves both an edge and top pulling. The dashed black line corresponds to the F_S value prior to precursor nucleation: the corresponding shape of $|\sigma(x, z_P)|$ is shown in Fig.7(a) (red curve). (b) Internal average shear stress obtained from Eqs.S71-S86 and averaged along the y direction, i.e. $|\tau(x, z_P)|$. The dashed black line corresponds to the F_S value prior to precursor nucleation: the corresponding shape of $|\tau(x, z_P)|$ is shown in Fig.7(a) (blue curve). $F_N = 6.25$kN, $L_x = 200$mm, $L_y = 7$mm, $L_z = 100$mm, $h = 6$mm ($\Delta h = 2$mm). Plots are averaged over 10 independent realizations.

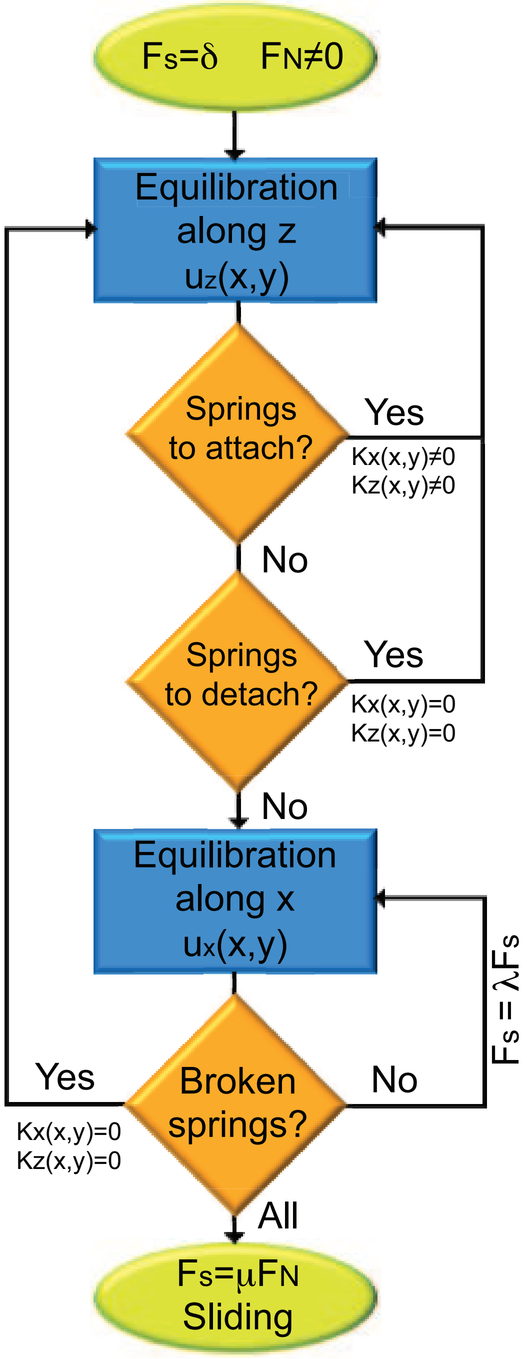

FIG. S11. **Scalar model algorithm**. Flowchart of the quasi-static dynamical protocol of the scalar model.

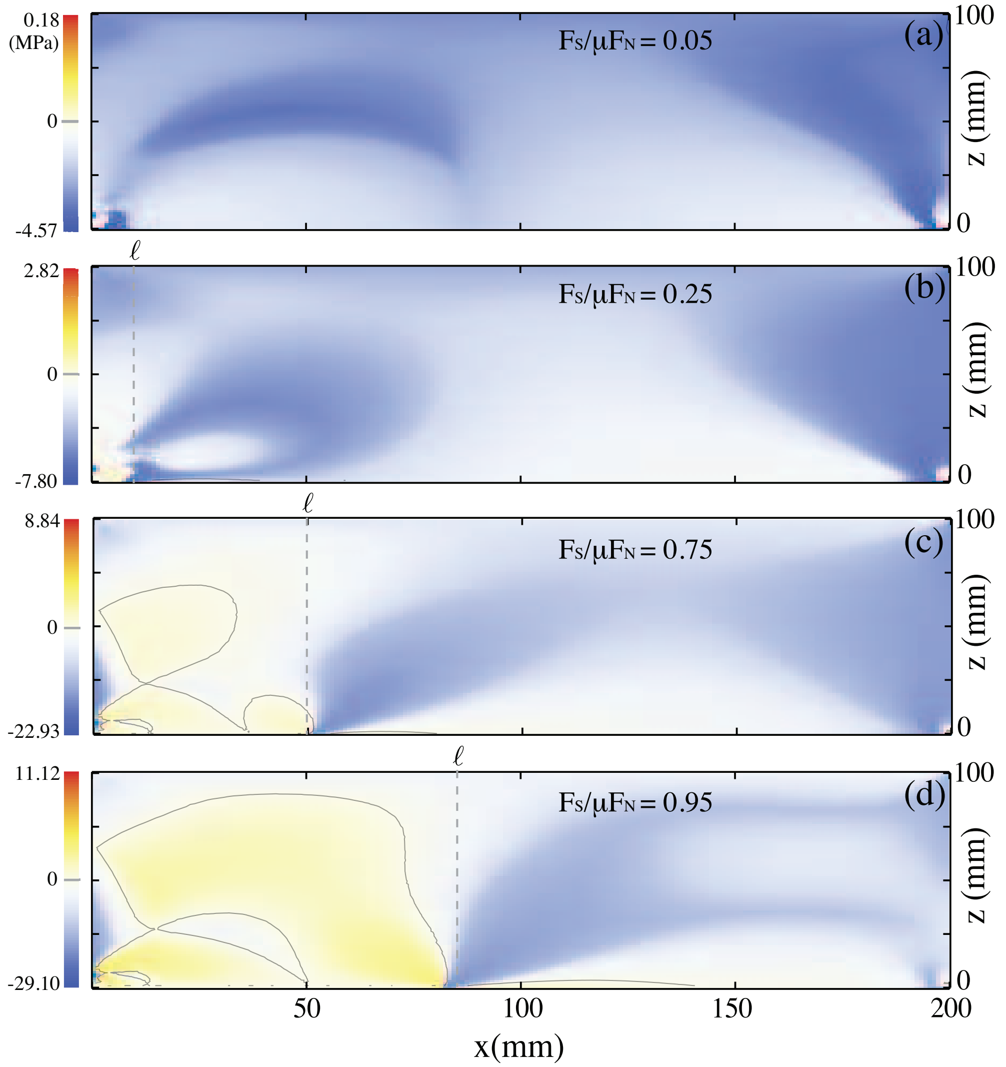

FIG. S12. **Coulomb stress quasi-static evolution in FEM**. (a)-(d) Quasi-static evolution of the Coulomb stress (calculated at $y = L_y/2$) along the slab $x - z$ plane. Color code indicates regions where $\tau_C > 0$ (yellow-red) from those for which $\tau_C < 0$ (blu), grey solid lines correspond to the set of points fulfilling $\tau_C = 0$. The loading conditions are the same as Fig.8 and the area of the detached contact surface is $\ell \times L_y$, where ℓ is the corresponding precursor size achieved in the scalar model.

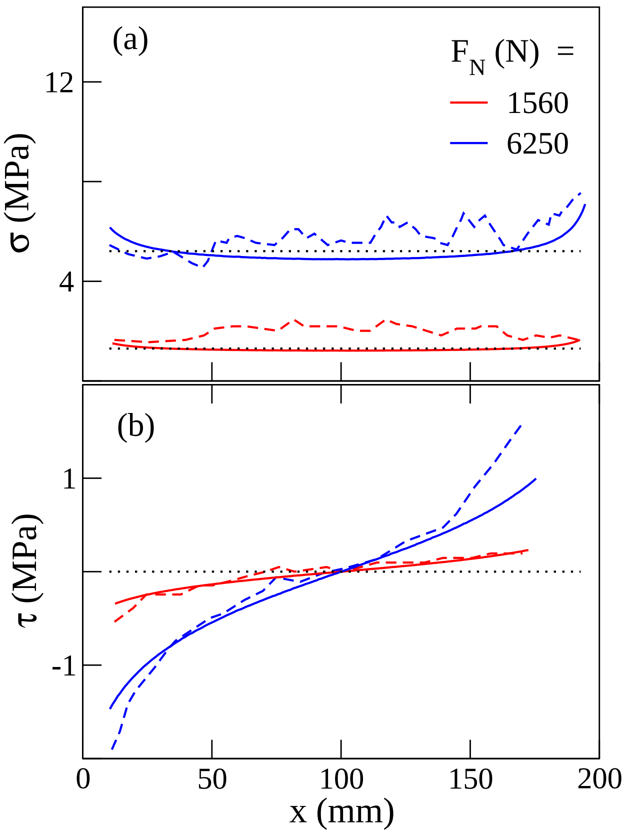

FIG. S13. **FEM model: calibration and stress profiles**. (a)-(b) Calibration of the model by comparison with (a) normal and (b) shear stress profiles of the experiment reported in [10] (dashed lines), calculated on the set of points $(x, y = L_y/2, z = z_p = 2mm)$. No shearing force is applied, and $L_x = 200$mm, $L_y = 6$mm, $L_z = 100$mm. Solid lines represent the outcomes of FEM simulations after setting the interfacial asperities stiffnesses to $k_x = 10^5$MPa/m and $k_z = 10^6$MPa/m. The model shows good agreement with the experiments, for both values of the applied normal force F_N. Dotted black lines symbolize normal and shear internal stresses calculated by the scalar model: the stresses appear to be uniform ($\sigma = F_N/(L_x L_y)$, $\tau = 0$) since no Poisson expansion and corner divergences are encompassed (interfacial rough fluctuations are averaged out).

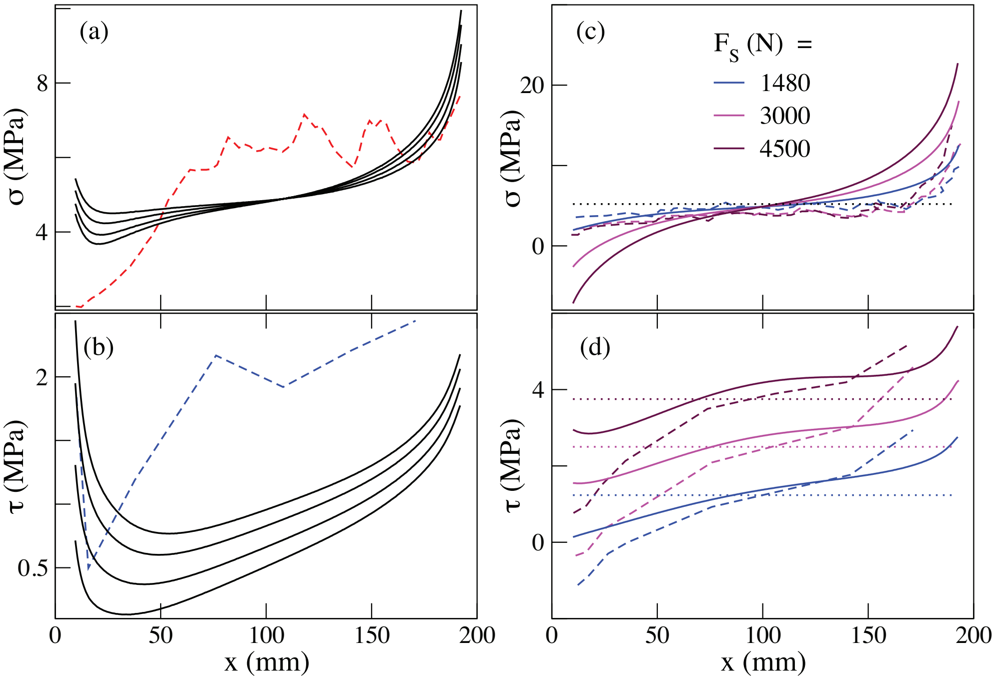

FIG. S14. **FEM shearing: internal stress profiles** . Normal (a) and shear (b) internal stresses calculated on the set of points $(x, y = L_y/2, z = z_p = 2mm)$, when the shear force is applied both at the trailing edge and uniformly on top of the PMMA sample ($F_s = F_s^{top} + F_S^{lat}$). Sample dimensions are $L_x = 200$mm, $L_y = 6$mm, $L_z = 100$mm and the normal load is $F_N = 6250$N. The experimental curves (dashed lines) cannot be reproduced by the FEM model, whose stress profiles are represented by black solid lines for values of $F_s = F_s^{top} + F_s^{lat} = 1500$N, 1300N, 1040N and 800N from top to down ($F_s^{top} = F_s^{lat}$). (c) and (d) show the normal and shear internal stresses when shear force is applied uniformly on top of the sample, i.e. $F_S = F_S^{top}$: solid lines represent the outcomes of FEM simulations and dashed lines stand for the experimental curves (extracted from Fig.1B-C of Ref.[10]). Although the FEM model captures the trend, the agreement between numerics and experiments is not satisfactory (although slightly better than lateral shearing, in panels (a) and (b)). Dotted lines represent the normal and shear stress profiles calculated according to the scalar model. In this case the stresses appear uniform as a consequence of the decoupling of the displacements u_x and u_z (scalar elasticity approximaton).
